# Primary care clinics can be a source of exposure to virulent *Clostridium* (now *Clostridioides*) *difficile*: An environmental screening study of hospitals and clinics in Dallas-Fort Worth region

**DOI:** 10.1371/journal.pone.0220646

**Published:** 2019-08-15

**Authors:** Jerry W. Simecka, Kimberly G. Fulda, Mark Pulse, Joon-hak Lee, John Vitucci, Phung Nguyen, Patricia Taylor, Frank Filipetto, Anna M. Espinoza, Sushma Sharma

**Affiliations:** 1 Department of Pharmaceutical Sciences and UNT Preclinical Services, University of North Texas System College of Pharmacy, University of North Texas Health Science Center, Fort Worth, TX, United States of America; 2 Texas College of Osteopathic Medicine, University of North Texas Health Science Center, Fort Worth, TX, United States of America; 3 The North Texas Primary Care Practice-Based Research Network (NorTex), University of North Texas Health Science Center, Fort Worth, TX, United States of America; 4 Department of Biostatistics and Epidemiology, School of Public Health, University of North Texas Health Science Center, Fort Worth, TX, United States of America; 5 The Dallas-Fort Worth Hospital Council Education and Research Foundation, Irving, TX, United States of America; Cornell University, UNITED STATES

## Abstract

*C*. *difficile* is an endospore-forming pathogen, which is becoming a common cause of microbial health-care associated gastrointestinal disease in the United States. Both healthy and symptomatic patients can shed *C*. *difficile* spores into the environment, which can survive for long periods, being resistant to desiccation, heat, and disinfectants. In healthcare facilities, environmental contamination with *C*. *difficile* is a major concern as a potential source of exposure to this pathogen and risk of disease in susceptible patients. Although hospital-acquired infection is recognized, community-acquired infection is an increasingly recognized health problem. Primary care clinics may be a significant source of exposure to this pathogen; however, there are limited data about presence of environmental *C*. *difficile* within clinics. To address the potential for primary care clinics as a source of environmental exposure to virulent *C*. *difficile*, we measured the frequency of environmental contamination with spores in clinic examination rooms and hospital rooms in Dallas-Fort Worth (DFW) area of Texas. The ribotypes and presence of toxin genes from some environmental isolates were compared. Our results indicate primary care clinics have higher frequencies of contamination than hospitals. After notification of the presence of *C*. *difficile* spores in the clinics and an educational discussion to emphasize the importance of this infection and methods of infection prevention, environmental contamination in clinics was reduced on subsequent sampling to that found in hospitals. Thus, primary care clinics can be a source of exposure to virulent *C*. *difficile*, and recognition of this possibility can result in improved infection prevention, potentially reducing community-acquired *C*. *difficile* infections and subsequent disease.

## Introduction

*Clostridium* (now *Clostridioides*) *difficile*-associated disease (CDAD) is a leading cause of gastroenteritis associated deaths [1, 2] and becoming the most common microbial cause of health-care associated gastrointestinal disease in the United States [2, 3]. Health care costs due to *C*. *difficile* infections are estimated to be about $5 billion annually [4]. *C*. *difficile* is an endospore-forming pathogen that can affect the gastrointestinal system primarily in at-risk patients [5]. Both asymptomatic and symptomatic patients can shed *C*. *difficile* spores into the environment, which are resistant to desiccation, heat, and various disinfectants [6, 7]. The spores can reside in the gut of healthy individuals and not germinate while in the presence of an intact and healthy gut microflora, but in some circumstances germinated to vegetative (growing) cells. Production of exotoxins by vegetative *C*. *difficile* results in disruption of normal epithelial function and potentially severe diarrhea, leading to hospitalization and mortality, especially in elderly or immunocompromised patients.

Environmental contamination of health-care facilities with *C*. *difficile* spores is a major concern in the transmission of this pathogen. Hospitals are recognized as sites for exposure to this pathogen and subsequent development of disease [7, 8]. However, community-acquired *C*. *difficile* disease occurs in patients who have not been recently admitted to health care facilities (i.e. 90 days) and is becoming recognized as a significant problem, despite an unclear source of acquisition [9, 10]. It has been suggested that outpatient care clinics are a significant source of community exposure to this pathogen [11], but further studies are needed to determine if primary care clinics have a similar potential for exposure of patients as found in the hospital environment.

Environmental and clinical isolates of *C*. *difficile* can vary in their potential to infect and cause disease. Virulence of *C*. *difficile* is linked to the expression of toxin genes [12], as well as known ribotypes used to identify epidemic/hypervirulent isolates [13–15]. The development of *C*. *difficile* disease is dependent upon the expression of toxins A (TcdA) and B (TcdB) [12, 16, 17]. These glycosylating toxins damage the intestinal epithelium, leading to inflammation and diarrhea. A third toxin, binary toxin (CDT), has also been identified in 17% to 23% of *C*. *difficile* strains, but it is currently unclear whether CDT plays a significant role in disease pathogenesis [18–20]. Additionally, there are multiple ribotypes of *C*. *difficile*, which are often identified in epidemiological studies for the assessment of environmental transmission and potential virulence. In North America, the most prevalent epidemic strain of *C*. *difficile* is BI/NAP1/027 or ribotype 027 [13, 15], but other ribotypes are also found as a cause of disease, including ribotype 078 [21–25]. Importantly, there may be differences in virulence associated with different ribotypes of *C*. *difficile* [26]. In addition to clinical evidence, ongoing studies in our lab have shown that epidemic ribotypes, like 027, are more virulent in animal models than non-epidemic isolates (J.W. Simecka, Personal communication). Thus, ribotyping in addition to the presence of toxin genes can be used to assess the potential virulence and epidemiology of *C*. *difficile* isolates.

To address the potential for primary care clinics as a source of environmental exposure to virulent *C*. *difficile*, we measured the frequency of environmental contamination with spores in clinic examination rooms in Dallas-Fort Worth (DFW) area of Texas and compared them with hospitals in the same area of Texas. The ribotypes and presence of toxin genes from a portion of the environmental isolates were also determined and compared. In addition, procedures to disinfect hospital rooms from environmental contamination with *C*. *difficile* spores can be inadequate [27], and it is possible that cleaning procedures can be improved in primary care clinics. Thus, we examined whether disclosure of the presence of spores impacted subsequent attempts to detect spores in the environment. In clinics, we not only provided this information but also included an educational discussion to emphasize the importance of this infection and methods of infection prevention to prevent transmittance of environmental *C*. *difficile*. Our results indicate indeed primary care clinics have higher frequencies of contamination than that found in hospitals, but after notification and education, environmental contamination in clinics was reduced to that found in hospitals. Thus, primary care clinics can be a significant source of exposure to virulent *C*. *difficile*, and recognition of this possibility can result in improved infection prevention, potentially reducing community-acquired *C*. *difficile* infections and subsequent disease.

## Materials and methods

### Ethics statement

This University of North Texas Institutional Review Board determined that this project did not meet the definition of human subject research, and the project was exempt from IRB review. Since the IRB determined this study did not include human subject research, informed consent was not required or obtained.

### Health care facilities

A total of 33 healthcare facilities in Texas were recruited to determine if *C*. *difficile* is detected through environmental sampling of patient/examination rooms. Three hospitals chose to withdraw from the study after the first sampling day because of their participation in a similar study. Out of the 30 remaining facilities, the 19 hospitals and 11 Family Medicine clinics were sampled. Of the 11 clinics, 5 were located in rural areas making up 18.2% of the total healthcare facilities. Most of the sampling was done in the inner city of an urban area (36.4%). The numbers and types of facilities were determined by the Dallas Fort Worth Hospital Council Foundation, as stated in the Hospital Engagement Network (HEN) *Clostridium difficile* (C-Diff) Environmental Research Project; power analysis was not calculated prior to these studies.

Environmental samples of each facility were collected over two rounds. Within each round, each facility was visited three times with approximately one week between each visit. The hospital rooms were all on the general care floors and did not include intensive care units. For each visit, seven sites (exam table/bed rails, doorknob, keyboards, light switches, restroom sink handles and faucet, toilet handles/pushbutton, and window blind wands/curtain) within a single patient/examination room were sampled. After the first round, each facility was provided the results of the environmental screening. For clinics, we also included an informational/educational discussion to emphasize the importance of *C*. *difficile* infection and methods of infection prevention to prevent transmittance of environmental *C*. *difficile* after the first round of sampling. The hospitals elected to not participate in the additional education session. All facilities maintained their standard cleaning processes, and sampling was performed on rooms cleaned, processed and ready for a patient; the timing of prior cleaning to sampling was not studied. The first round of sampling began on June 25^th^, 2014 and ended on August 22^nd^, 2014. The second round of sampling began on September 29^th^, 2014 and ended on November 24^th^, 2014.

### Survey

A 16-question survey, developed by the research team, was completed by personnel at each site during the first sample collection (S1 File). The survey included questions about site specific cleaning practices/policies and patient demographic information. Specifically, sites were asked if they had guidelines for *C*. *difficile* infection prevention and what their cleaning practices were for each of the seven sites that were sampled for surface-associated contamination. An infection preventionist generally completed the survey for the hospitals; while, an administrative director generally completed the survey for the primary care clinics.

### Environmental sampling

Wet wiping with sterile cloths (Swiffer TM, Proctor and Gamble) were used to collect environmental samples in each of the healthcare facilities. Within a patient or examination room at a healthcare facility, there were seven sites sampled during each visit. These sites included: Light switches, doorknobs, window blind wands/curtains, toilet handles/pushbutton, restroom sink handles and faucet, keyboards, and exam table/bed rails. When the rooms did not have restrooms located within the room, the nearest restroom was used for sampling. The same procedure was used for keyboards if one was not located in the room. If there were no window blind wands/curtains in the room, then the opthalmoscope/otoscope handles or the chair in the room was sampled. In addition to these seven sites within the examination room, a negative control was used to ensure that the sampler was not carrying *C*. *difficile* on their hands. Sterile gloves were worn during sampling and were changed between each sampling site. Each of the individuals collecting the environmental samples were trained on best procedures on using and removing personal protective equipment, including sterile gloves. Prior to sampling, hand hygiene was performed but not between changing of gloves.

### Initial isolation and identification of environmental *C*. *difficile*

Samples were processed similarly to previous studies [28, 29]. The bags containing the sampling cloths were transferred into a Don Whitley A35 or DG250 anaerobic workstation (Microbiology International, Fredrick, MD), and 30 mL of reduced brain heart infusion broth supplemented with 0.5% taurocholate (BHI-TA) was added into each bag containing the sampling cloth. After 5 days anaerobic incubation in the workstation at 37°C, 2 mL of the culture was removed and transferred to a sterile 2-mL Eppendorf tube. The sample was centrifuged for 5 minutes at 8,000x*g*, and the pellet was re-suspended in 2 mL of 70% ethyl alcohol for 1 hour and centrifuged again for 5 minutes at 8,000x*g*. The pellet was re-suspended in 0.2 mL of reduced BHI-TA and spread onto reduced cycloserine-cefoxitin-fructose agar plates (CCFA) containing 0.1% (w:v) sodium taurocholate, which is selective for growth of *C*. *difficile*. A positive control (0.2 mL of prepared spores from *C*. *difficile* BAA-1875 inoculated into a sterile sample bag containing a PBS saturated Swiffer cloth) was included with each set of environmental samples. After each plate had been incubated anaerobically at 37°C for 4 days, they were inspected for *C*. *difficile* growth. Positive *C*. *difficile* growth was identified as colonies with spreading morphologies, irregular margins, and a yellowing of the medium due to acid production during the fermentation of fructose by *C*. *difficile*. Colonies initially identified as *C*. *difficile* were spread onto CCFA agar and tryptic soy agar (TSA) containing 5% sheep’s blood and anaerobically incubated for 48 hours at 37°C. CCFA agar growth was evaluated for yellow-green fluorescence under long-wave UV light, and colonies from blood agar plates were confirmed as *C*. *difficile* using a simple latex agglutination assay (Oxoid Ltd, UK). Based on the phenotypic results of these tests, all presumed isolates of *C*. *difficile* were assigned a strain number and stored in cryogenic cultures at -80°C.

### *C*. *difficile* education session

The *C*. *difficile* education sessions took place after the first round of sampling. An infection preventionist, who was a part of the education team, attended meetings with clinic staff either in person or by phone and used a PowerPoint (Microsoft) presentation to provide background information and the definition of *C*. *difficile* (S2 File). Signs and symptoms of disease were reviewed in the presentation along with general methods to avoid *C*. *difficile*, such as, washing hands with soap and water, placing patients in isolation, cleaning with bleach, and antibiotic stewardship programs. The education provided to the participating locations was to provide awareness of the potential environmental exposure to *C*. *difficile* and disease. It was not specifically aimed at cleaning for *C*. *difficile*, but it did reference established cleaning guidelines for each facility. All participants were given brochures and posters to distribute and display to help increase the public’s awareness.

### Ribotyping of isolates

The ribotype of the 39 *C*. *difficile* isolates collected from various hospitals and clinics around DFW in the 2014 environmental study was performed by PCR analysis [30]. Control strains for the *C*. *difficile* ribotypes 027 and 078, previously obtained from American Type Culture Collection (ATCC) and characterized in the Simecka lab, were also included in the study.

Primers 16S (5’-carboxyfluorescein (Fam) Dye-GTGCGGCTGGATCACCTCCT-3’) and 23S (5′-CCCTGCACCCTTAATAACTTGACC-3′) (Thermo-Fischer Scientific) were used in capillary electrophoresis polymerase chain reaction (PCR) ribotyping. These primers were described by Bidet *et*. *al*. [31]. DNA was extracted from cultures to a final volume of 20 μl using the High Pure Product DNA kit (Roche) according to manufacturer’s instructions. Amplification reactions contained 5 μl of Buffer II, 1 μl DNTP’s, 1 μl of forward and reverse primers as previous described, 31.75 μl water, 0.25 μl of Taq Polymerase, and 10 μl of sample DNA. Samples were amplified in a commercial PCR thermocycler running a 95°C initial step for enzyme activation followed by 35 cycles of 1 min at 95°C for denaturation, 1 min at 57°C for annealing and 1 min at 72°C for elongation, plus a 5 min 72°C final elongation step.

PCR fragments were analyzed in a Hitachi 3500xL genetic analyzer with a 36 cm capillary loaded with a POP4 gel (Applied Biosystems). The size of each peak was determined using Peak Scanner software (Applied Biosystems).

Peaks in Bioanalyzer were counted as bands when they showed at least 5% of the height of the highest peak of the individual run. Double peaks were counted only if they were separated by more than 1.5 base pairs (bp). A web-based database (http://webribo.ages.at) was crafted for capillary gel electrophoresis-based PCR ribotyping results. An error margin of ±4 bp was incorporated into the analysis algorithm of the database. Using this web-based database, all users are able to enter their own data and receive a ribotype identification for each submitted isolate.

### Detection of toxin A and B genes

PCR analysis was used to detect the presence of the toxin A (*tcdA*) and toxin B (*tcdB*) genes. For the toxin A gene, a set of oligonucleotides were utilized to amplify different regions of the toxin A gene found in *C*. *difficile*. Primers YT-28 (5′-GCATGATAAGGCAACTTCAGTGG-3′) and YT-29 (5′-GAGTAAGTTCCTCCTGCTCCATCAA-3′) were designed by Y.J. Jang et. al. (1998) to amplify a region of the toxin A gene. For the toxin B gene, primers NK-104 (5′- GTGTAGCAATGAAAGTCCAAGTTTACGC-3′) and NK-105 (5′- CACTTAGCTCTTTGATTGCTGCACC-3′), as described by H. Kato et. al. (1998), were used to amplify a non-repeating portion of the toxin B gene. The DNA samples from the 39 isolates described above were amplified in 2.5 μl of Buffer II, 0.5 μl DNTP’s, 0.5 μl of forward and reverse toxin specific primer pair, 15.5 μl water, 0.5 μl of Taq Polymerase, and 5 μl of sample DNA. Samples were amplified in a commercial PCR thermocycler running a 95°C initial step for 2 minutes followed by 35 cycles of 45 seconds at 95°C for denaturation, 30 min at 55°C for annealing and 45 seconds at 70°C for elongation. The PCR results were analyzed by Experion 1K DNA chips read on an Experion Bioanalyzer (Bio-Rad). The chips were set-up with the included reagents (DNA stain and DNA 1K gel, ladder, and loading buffer) and then ran according to included manufacturer’s instructions. Isolates or control strains positive for the toxin A gene showed amplification at a size between 630–640 bp. Whereas, those that were positive for the toxin B gene showed amplification at a size between 230–240 bp.

### Statistical analyses

Data were evaluated by Fisher’s exact test or unpaired t test. A *p* value ≤ 0.05 was considered statistically significant. Two-tailed tests were used to determine whether there were differences between groups, while one-tail tests were used to determine if there was a reduced frequency of *C*. *difficile* after notification of results and education information was given to the facilities. Analyses were performed using JMP 10 (SAS Institute, Cary, NC) or Prism software (Graphpad Software, La Jolla, CA).

## Results

### Infection prevention survey of clinics and hospitals prior to sampling for environmental *C*. *difficile*

Data were collected for 19 hospitals and 11 Family Medicine clinics. Of the clinics, five were located in rural Texas with two of those clinics serving a community with less than 2,500 people. Five (45.5%) of the 11 clinics were solo practices. Six (54.5%) clinics and 19 (100.0%) hospitals reported having a policy on infection prevention; while, five (45.5%) clinics and 19 (100.0%) hospitals reported that they facilitate training/education to staff on infection prevention. Only one (9.1%) clinic said they have specific guidelines for *C*. *difficile* infection prevention compared to all 19 (100.0%) of the hospitals.

Responses to the question “How often does your clinic/hospital clean (disinfect) these surfaces” are provided in Table 1. Respondents reported that most hospital sites were cleaned every day or every week. The exceptions included two hospitals for which keyboards were cleaned every month and one hospital for which window blind wands/curtains were cleaned every six months. More variation in cleaning practices was reported for clinics. Exam tables/bedrails were never cleaned for three clinics, and doorknobs were never cleaned for one clinic. Additionally, light switches, window blind wands/curtains, and keyboards were never cleaned for two clinics.

**Table 1:** Results from survey question, “How often does your clinic/hospital clean (disinfect) these surfaces?”.

Hospitals						
Surfaces	every day	every week	every month	every 6 months	never	missing
light switches	15 (78.9%)	2 (10.5%)	0	0	0	2 (10.5%)
door knobs	15 (78.9%)	2 (10.5%)	0	0	0	2 (10.5%)
window blind wands / curtains	7 (36.8%)	6 (31.6%)	0	1 (5.3%)	0	5 (26.3%)
restroom commodes	18 (94.7%)	1 (5.3%)	0	0	0	0
sink handles	18 (94.7%)	1 (5.3%)	0	0	0	0
keyboards	15 (78.9)	1 (5.3%)	2 (10.5%)	0	0	1 (5.3%)
bedrails	17 (89.5%)	0	0	0	0	2 (10.5%)
Clinics						
Surfaces	every day	every week	every month	every 6 months	never	missing
light switches	3 (27.3%)	6 (54.5%)	0	0	2 (18.2%)	0
door knobs	3 (27.3%)	7 (63.6%)	0	0	1 (9.1%)	0
window blind wands / curtains	1 (9.1%)	3 (27.3%)	3 (27.3%)	0	2 (18.2%)	2 (182%)
restroom commodes	9 (81.1%)	2 (18.2%)	0	0	0	0
sink handles	9 (81.1%)	1 (9.1%)	0	0	0	1 (9.1%)
keyboards	3 (27.3%)	4 (36.4%)	1 (9.1%)	0	2 (18.2%)	1 (9.1%)
bedrails	3 (27.3%)	1 (9.1%)	0	0	3 (27.3%)	4 (36.4%)

### Environmental contamination with *C*. *difficile* was common in clinics

Although hospital-acquired *C*. *difficile* infections are well recognized [8, 32], recent studies suggest that the presence of *C*. *difficile* spores within the environment of clinics is a source of community-acquired *C*. *difficile* infections [32, 33]. In our study, all facilities maintained their standard cleaning processes, and sampling was performed on rooms cleaned, processed and ready for a patient. After processing samples and verifying results, *C*. *difficile* environmental isolates were recovered more frequently from clinics than hospitals (Table 2). Environmental samples from ten out of the 11 clinics tested had at least one positive sample, while five out of the 19 hospitals tested had *C*. *difficile* recovered from environmental samples (Fisher’s exact test, p ≤ 0.05). However, clinic and hospital facilities where environmental *C*. *difficile* were recovered had a similar percentage (7.6%) of positive samples (unpaired t test, no significant difference). Thus, clinics were a frequent source of potential exposure to *C*. *difficile*, even more commonly contaminated than hospital rooms.

**Table 2:** Prevalence of *C*. *difficile* in samples obtained from participating health care facilities prior to information sessions.

Clinics			Hospitals		
Facility	No. Positive Samples	Percentage (Out of 21 total samples)	Facility	No. Positive Samples	Percentage (Out of 21 total samples)
C012	1	4.76	H001	1	4.76
C013	4	19.05	H002	0	0.00
C014	1	4.76	H003	1	4.76
C015	3	14.29	H004	0	0.00
C016	1	4.76	H005	0	0.00
C017	1	4.76	H006	0	0.00
C022	1	4.76	H007	1	4.76
C023	2	9.52	H008	2	9.52
C024	1	4.76	H009	0	0.00
C025	0	0.00	H010	0	0.00
C032	1	4.76	H011	0	0.00
			H021	0	0.00
			H026	0	0.00
			H027	0	0.00
			H028	0	0.00
			H029	0	0.00
			H030	0	0.00
			H031	0	0.00
			H033	3	14.29
	Frequency of Positive Clinics	% positive samples from positive facilities^a^		Frequency of Positive Hospitals	% positive samples from positive facilities
Summary	10 out of 11 facilities*	7.6 (5.1)		5 out of 19 facilities	7.6 (4.2)

^a^Mean (± SD) of number of samples from individual facilities where *C*. *difficile* was recovered.

*There was a higher frequency of clinics where *C*. *difficile* was recovered than hospitals (p ≤ 0.05, Two tailed Fischer’s exact test).

In both clinics and hospitals, the primary sites where *C*. *difficile* was recovered included bed rails or examination bed and doorknobs (Table 3). In hospitals, restroom sink handles and faucets were also commonly contaminated; whereas in clinics, keyboards were a major site for *C*. *difficile* recovery. Thus, it appears that locations in rooms that are often touched by hands of patients and staff are more often potential sources of transmission.

**Table 3:** *C*. *difficile* prevalence by type of health care facilities and sampling site.

	Type of health care facility		
	Clinics		
Sampling site	No. of samples tested^a^	No. positive samples	%
Exam table	33	6	18.18
Doorknob	33	2	6.06
Keyboards	33	5	15.15
Light switches	33	0	0.00
Restroom sink handles & faucet	33	1	3.03
Toilet handles/ pushbutton	33	1	3.03
Window blind wands/curtain	33	1	3.03
Subtotal	231	16	6.93
			
	Hospitals		
Sampling site	No. of samples tested	No. positive samples	%
Bed rails	57	0	0.00
Doorknob	57	2	3.51
Keyboards	57	1	1.75
Light switches	57	0	0.00
Restroom sink handles & faucet	57	3	5.26
Toilet handles/ pushbutton	57	1	1.75
Window blind wands/curtain	57	1	1.75
Subtotal	399	8	2.01

^a^Clinics had 3 samples per site per facility (11 clinics); hospitals had 3 samples per site per facility (19 hospitals).

### Increased education reduced the frequency of spore recovery in clinics

After the first round of environmental sampling, each of the healthcare facilities was provided the results of the testing. The facilities were offered an additional educational seminar for the staff that emphasized the impact of *C*. *difficile* (Supplemental information) on healthcare and the importance of infection prevention. The hospitals did not accept the offer for additional training; whereas, all of the clinics did. After the first round of sampling, notification of results, and educational seminar (if given), a second round of environmental sampling was performed. As in the first round, each of the facilities were visited on three different dates and sampling of seven sites within an empty patient or examination room was performed during each visit (Table 4).

**Table 4:** Prevalence of *C*. *difficile* in samples obtained from participating health care facilities after the information sessions and/or notification of results from first round of sampling.

Clinics			Hospitals		
Facility	No. Positive Samples	Percentage (Out of 21 total samples)	Facility	No. Positive Samples	Percentage (Out of 21 total samples)
C012	0	0.00	H001	0	0.00
C013	1	4.76	H002	3	14.29
C014	0	0.00	H003	1	4.76
C015	1	4.76	H004	2	9.52
C016	4	19.05	H005	1	4.76
C017	0	0.00	H006	0	0.00
C022	7	33.33	H007	0	0.00
C023	0	0.00	H008	2	9.52
C024	0	0.00	H009	0	0.00
C025	0	0.00	H010	0	0.00
C032	1	4.76	H011	3	14.29
			H021	0	0.00
			H026	2	9.52
			H027	5	23.81
			H028	0	0.00
			H029	3	14.29
			H030	0	0.00
			H031	0	0.00
			H033	0	0.00
	Frequency of Positive Clinics	% positive samples from positive facilities^a^		Frequency of Positive Hospitals	% positive samples from positive facilities
Summary After information sessions/notification	5 out of 11 facilities*	13.3 (12.8)		9 out of 19 facilities	11.6 (5.9)
Summary (Table 1) Before information sessions/notification	10 out of 11 facilities*	7.6 (5.1)		5 out of 19 facilities	7.6 (4.2)

^a^Mean (± SD) of number of samples from individual facilities where *C*. *difficile* was recovered.

*There was a lower frequency of clinics where *C*. *difficile* was recovered after the information session, than prior to these sessions (p ≤ 0.05, One tailed Fischer’s exact test). There was no difference found in the frequency of *C*. *difficile* recovery in hospitals due to notification of results.

There was a difference in the frequency of *C*. *difficile* positive rooms in clinics, but not hospitals after notification and/or education of environmental contamination in these facilities. Prior to education and notification of culture results, *C*. *difficile* was found in 10 out of 11 clinics, but afterwards, only five out of the 11 clinics were positive. Thus, there was a significant reduction (Fisher’s exact test, p ≤ 0.05) in the frequency of environmental contamination in the clinics. In contrast, there was no overall difference between the frequency of hospitals that were positive before (five out of 19) and after (nine out of 19) notification of culture results, although there may have been an effect on specific hospitals. Interestingly, there was no difference in the frequency of environmental contamination between clinics and hospitals after the education of clinic staff (Fisher’s exact test, p ≤ 0.05).

### Ribotypes and presence of toxin genes were similar in isolates from hospitals and clinics

Environmental isolates of *C*. *difficile* can vary in their potential to infect and cause disease. To further compare the isolates from clinic and hospitals, 20 different clinic isolates and 19 different hospital isolates were characterized for their ribotype and presence of toxin genes. Both ribotyping and presence of toxin genes can be used to assess the potential virulence of *C*. *difficile* isolates, and by comparing those results among the environmental isolates, the likelihood of environmental contamination of clinics being a source of community-acquired disease can be evaluated.

Six out of ten clinics had isolates with similar *C*. *difficile* ribotypes to that found in hospitals, indicating that a similar profile of isolates can be found in both hospitals and clinics (Table 5). Interestingly, ribotype 078 isolates were found in 3 clinic or hospital facilities. Importantly, *C*. *difficile* ribotype 078 has been most frequently found in animals, but the 078 ribotype has been characterized as hypervirulent and increasingly found as a cause of CDAD in humans [15, 34, 35]. In contrast, 027 ribotype is an epidemic strain found in North America [15, 36], and only one hospital isolate, and none of the clinical isolates, was found to be this ribotype.

**Table 5:** Ribotypes of environmental *C*. *difficile* isolates collected from clinics and hospitals.

Type of facility	Facility	Common ribotype*	Unique ribotype
Clinics	C012		707
	C013	078	066
	C014		241
	C015	078	039
	C016	078, AI83	AI58
	C017		699
	C022	413	
	C023	063	441
	C024		AI60
	C032	552	
Hospitals	H001	552	
	H003	078, AI83	
	H004	AI83	027
	H005		626
	H007		001 ecdc
	H008	413	582
	H011	078	218
	H026	AI83	693
	H029	063	
	H033	078	

*Common ribotypes refer to ribotypes found in both clinics and hospitals, while unique ribotypes are those found only in one site. Ribotypes 027 and 078 are identified as epidemic and/or more virulent ribotypes [15, 34–36]

About 90% of the isolates from clinics and hospitals had the genes encoding either toxin A or toxin B. In one clinic (C016), two isolates (ribotype AI58) had the toxin B gene but not the toxin A gene, but other isolates from that clinic had both toxin genes. There were some isolates that did not have either of the toxin genes (1/20 clinic isolates; 2/19 hospital isolates). Two isolates (ribotype 413) from a hospital (H008) had neither toxin A nor toxin B genes, but other isolates from that hospital had both genes. Additionally, a single isolate (ribotype 413) from one clinic (C022) did not have either toxin gene as well.

## Discussion

In healthcare facilities, environmental contamination with *C*. *difficile* spores is a major concern as this is a potential source of exposure to this pathogen and risk of disease in susceptible patients [37]. Although hospital-acquired infection is a known problem [22, 33], community-acquired CDAD, where patients acquire the disease in the absence of recent hospital admission, is also becoming increasingly recognized. Primary care clinics may be a significant source of exposure to this pathogen [22, 33], but there are limited data about presence of environmental *C*. *difficile* spores within clinics. To examine this possibility, the current study compared the presence of *C*. *difficile* spores in clinic examination rooms and hospital rooms in Dallas/Fort Worth area in Texas that were cleaned, processed and ready for a patient.

*C*. *difficile* was recovered from both clinic examination rooms and hospital rooms that were ready for a patient. Importantly, all facilities maintained their standard cleaning processes, and sampling was performed on rooms cleaned, processed and ready for a patient. During the first round of sampling, *C*. *difficile* was recovered more frequently from clinics than from hospitals. However, there was no difference in the percentage of samples between a “contaminated” clinic and hospital, indicating a similar level of environmental contamination. *C*. *difficile* was reported on the hands of nurses after handling a patient with *C*. *difficile* in previous research [38]. In both clinics and hospitals sampled in the current study, the primary sites contamination included locations often touched by the hands of patients and/or staff, indicating a potential source of exposure to and spread of *C*. *difficile* [37]. Analysis of the ribotype distribution among the environmental isolates from clinics and hospitals show a commonality between the two types of health care facilities. As hospitals are associated with the spread of hospital-acquired *C*. *difficile* infections [8, 32], it is highly likely that clinics are a source of exposure to similar ribotypes of the pathogen. Different ribotypes may be more virulent than others [39] (J.W. Simecka, Personal communication). Furthermore, the presence of toxin A and B genes in most of the isolates from either clinic or hospital environments is supportive that the isolates were of similar virulence potential. Based on these results and recognition that environmental exposure can lead to hospital-acquired infections [32, 37], there is clearly the potential for clinics to be a significant source of community exposure to *C*. *difficile* and subsequent development of disease from infection, especially in susceptible patients.

Most likely, infection prevention and cleaning procedures in clinics need to be reemphasized. The frequency of sites where *C*. *difficile* was recovered was compared prior to and after disclosure of the results from the first round of sampling of clinic and hospital rooms. In all of the clinic sites, an educational presentation emphasizing the importance of CDAD and infection prevention was given. Although hospitals were given this option, none accepted. As described above, primary care clinics had higher frequencies of contamination than found in hospitals, but after the educational presentation, the frequency of *C*. *difficile* recovery in clinics was reduced to that found in hospitals. These results suggest that notification of environmental contamination and its potential impact influenced the efficiency of infection prevention procedures in clinics. However, *C*. *difficile* spores are resistant to heat, dehydration and many detergents [6, 7], and thus, in addition to improved hygiene practices by staff and clinicians, better cleaning procedures that inactivate *C*. *difficile* spores may further reduce potential exposure to patients in clinics and hospitals.

Overall, clinics are likely a significant source of community-acquired *C*. *difficile* infection and subsequent disease. Clinics were a frequent source of potential environmental contaminants with *C*. *difficile* spores, even more often than in hospital rooms. Most of environmental isolates from clinics and hospitals had toxin genes and overlapping ribotypes, indicating similar virulence potentials. This suggests that both hospital rooms and primary care clinics could be a potential source of exposure and subsequent development of *C*. *difficile* infections in susceptible individuals. There still may be differences in antibiograms of isolates obtained from hospitals and clinics, which would be consistent with the profiles of isolates from patients with hospital-acquired and community-acquired infections [40]. The current study indicates that better cleaning procedures should reduce environmental contamination of clinics, and that improved hand hygiene of healthcare workers would likely reduce the spread and contamination within health care facilities, including primary care clinics. Future studies examining the possible association of patients with *C*. *difficile* disease and visits to primary care clinics would provide further support about their role in impacting community exposure to *C*. *difficile*; however, the current study does provide compelling evidence that environmental contamination of virulent *C*. *difficile* are found in these clinics.

## Supporting information

S1 File Survey questions about site specific cleaning practices/policies. (DOCX)

S2 File PowerPoint presentation of *C*. *difficile* education sessions given to clinics. (PPTX)
