# Supplementary material for: Primary care clinics can be a source of exposure to virulent Clostridium (now Clostridioides) difficile: An environmental screening study of hospitals and clinics in Dallas-Fort Worth region
Source: PLoS One. 2019 Aug 15;14(8):e0220646. doi: 10.1371/journal.pone.0220646 (PMC6695158; doi:10.1371/journal.pone.0220646)
Supplement: S1 File — (DOCX) [file pone.0220646.s001.docx]

**Clostridium Difficile (C-Diff) Environmental Research Study and Education**

**Survey**

Kimberly Fulda, DrPH

Please call **(817) 735-0225** if you have any questions

The DFWHC Foundation and UNT Health Science Center are conducting a survey entitled the “Clostridium Difficile (C-Diff) Environmental Research Survey” which will allow researchers to learn about infection control policies and strategies in clinics and health care facilities. With this information, we will be able to facilitate educational programs and training to develop best practices to reduce C. Diff in your facility and sustainability of lower infection rates.

This survey is voluntary, and you are not required to answer all of the questions. By completing the survey, you are allowing DFWHC Foundation and UNT Health Science Center to use your responses for analysis purposes. All identifiable information will be kept confidential and will not be shared with anyone. Only blinded summary data of all responding facilities will be shared and used. Your answers to the survey will not affect your status and current relationship with either DFWHC Foundation or UNT Health Science Center. Please complete the survey and return it by mail or by fax to the address below.

We would greatly appreciate your participation in this survey.

Practice/ Hospital name: ________________________________________________________

Address: _____________________________________________________________________

Telephone Number: ____________________________________________________________

1. Please select the category that **best** describes your Organization Type:

- Government, Non-federal:
- Non-government, not-for-profit (NFP)
- Investor-owned, For-Profit
- Government, federal

1. Please select the category that **best** describes your primary practice location.

- ____ Inner City of urban area
- ____ Urban (not inner city)
- ____ Suburban
- ____ Rural

1. Please list the total number of beds in your organization (for hospitals only)?

- Less than 50 Beds
- 50–100 Beds
- 101–300 Beds
- 301–600 Beds
- 601–1,000 Beds
- 1,001+ Beds

1. Please select the category that **best** describes the *population* of the community in which your primary practice/ hospital is located.

- ____ Less than 2500 people
- ____ 2500 to 19,999 people
- ____ 20,000 to 249,999 people
- ____ 250,000 to 999,999 people
- ____ Greater than or equal top 1,000,000

1. Please select the category that **best** describes your practice type (use your primary clinic if you practice in more than one location).

- ____ Solo practice
- ____ Two-person partnership
- ____ Single specialty group (ie. Family medicine, internal medicine, etc)
- ____ Multispecialty group
- ____ Academic practice (residency program, faculty practice)
- ____ Other (please specify) _______________________________

1. Please share the total number of employees in 2013: ____________
2. Please share the total number of patients served in 2013? _________________

8. Please list the approximate number of ambulatory patient visits you have in an average week (include all practice sites).

a. Yourself ____

b. Entire practice/ hospital (if not solo practice) ____

c. Source of estimates for (EMR, billing data, best guess) ______________

9. Does your clinic/ hospital offer **any** of the following services?

a. Prenatal care ____Yes ____ No

b. Deliveries ____Yes ____ No

c. Postnatal care ____Yes ____ No

d. Colposcopy ____Yes ____ No

e. Flexible sigmoidoscopy ____Yes ____ No

f. Colonoscopy ____Yes ____ No

g. Treadmill testing ____Yes ____ No

h. Nasopharangoscopy ____Yes ____ No

i. Ultrasound scanning ____Yes ____ No

j. Hospital care ____Yes ____ No

k. Emergency room care ____Yes ____ No

10. Does your Clinic/ hospital have a policy on infection control?

- Yes
- No
- Do not know

11. Who oversees the infection control policy implementation?

- Clinical staff
- Administrative staff
- Do not have one

12. Does your clinic/ hospital facilitate training/ education to staff on infection control?

- Yes
- No
- Do not know

13. Does your clinic/ hospital have guidelines for staff for cleaning processes, personal hygiene and hand washing techniques, and antibiotic stewardship?

- Yes
- No
- Do not know

15. How often does your clinic/ hospital clean (disinfect) these surfaces (every day/every week/ every month/ every 6 months/ never)?

|  | Every day | Every Week | Every Month | Every 6  Months | Never |
| --- | --- | --- | --- | --- | --- |
| Light switches | 🞎 | 🞎 | 🞎 | 🞎 | 🞎 |
| Door Knobs | 🞎 | 🞎 | 🞎 | 🞎 | 🞎 |
| Window Blind wands/curtains | 🞎 | 🞎 | 🞎 | 🞎 | 🞎 |
| Restroom commodes | 🞎 | 🞎 | 🞎 | 🞎 | 🞎 |
| Sink Handles | 🞎 | 🞎 | 🞎 | 🞎 | 🞎 |
| Keyboards | 🞎 | 🞎 | 🞎 | 🞎 | 🞎 |
| Bedrails | 🞎 | 🞎 | 🞎 | 🞎 | 🞎 |

16. Does your clinic/hospital have any specific guidelines for C-Diff infection prevention?

- Yes
- No
- Do not know

If Yes, Please explain briefly ---------------------------------------------------------------------------------

If No, Would you like to learn more about C-Diff infection prevention for your clinic/hospital?

- Yes
- No
- Do not know

Thank you for completing this survey.

*Office Use Only*

Data Entry

1. Initials ______ Date In ______

2. Initials ______ Date In ______

*Office Use Only*

Data Entry

1. Initials ______ Date In ______

2. Initials ______ Date In ______

*Office Use Only*

Data Entry

1. Initials ______ Date In ______

2. Initials ______ Date In ______

*Office Use Only*

Data Entry

1. Initials ______ Date In ______

2. Initials ______ Date In ______

*Office Use Only*

Data Entry

1. Initials ______ Date In ______

2. Initials ______ Date In ______
