# Supplementary material for: Primary care clinics can be a source of exposure to virulent Clostridium (now Clostridioides) difficile: An environmental screening study of hospitals and clinics in Dallas-Fort Worth region
Source: PLoS One. 2019 Aug 15;14(8):e0220646. doi: 10.1371/journal.pone.0220646 (PMC6695158; doi:10.1371/journal.pone.0220646)
Supplement: S2 File — (PPTX) [file pone.0220646.s002.pptx]

## Slide 1
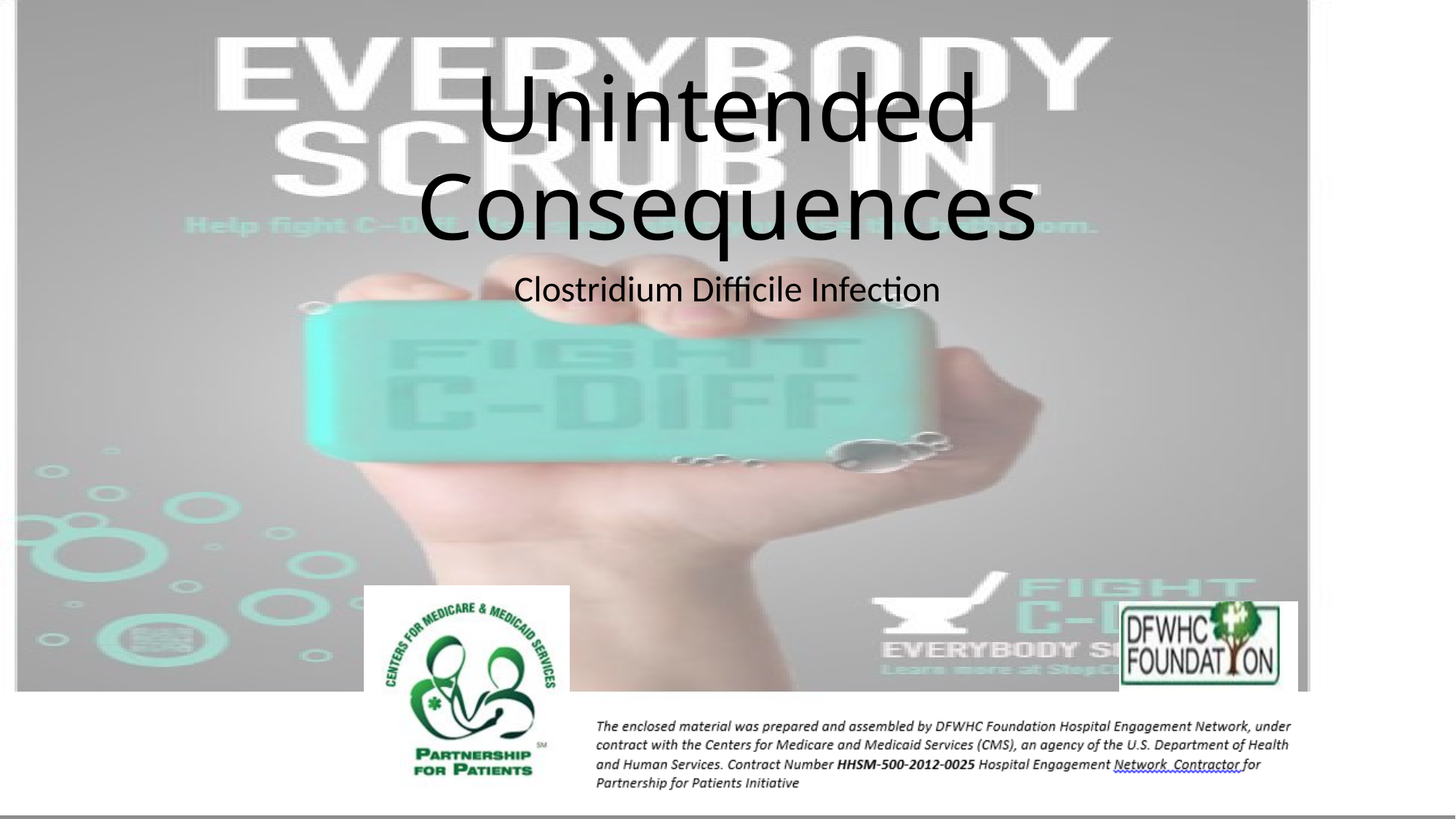

# Unintended Consequences
Clostridium Difficile Infection

## Slide 2
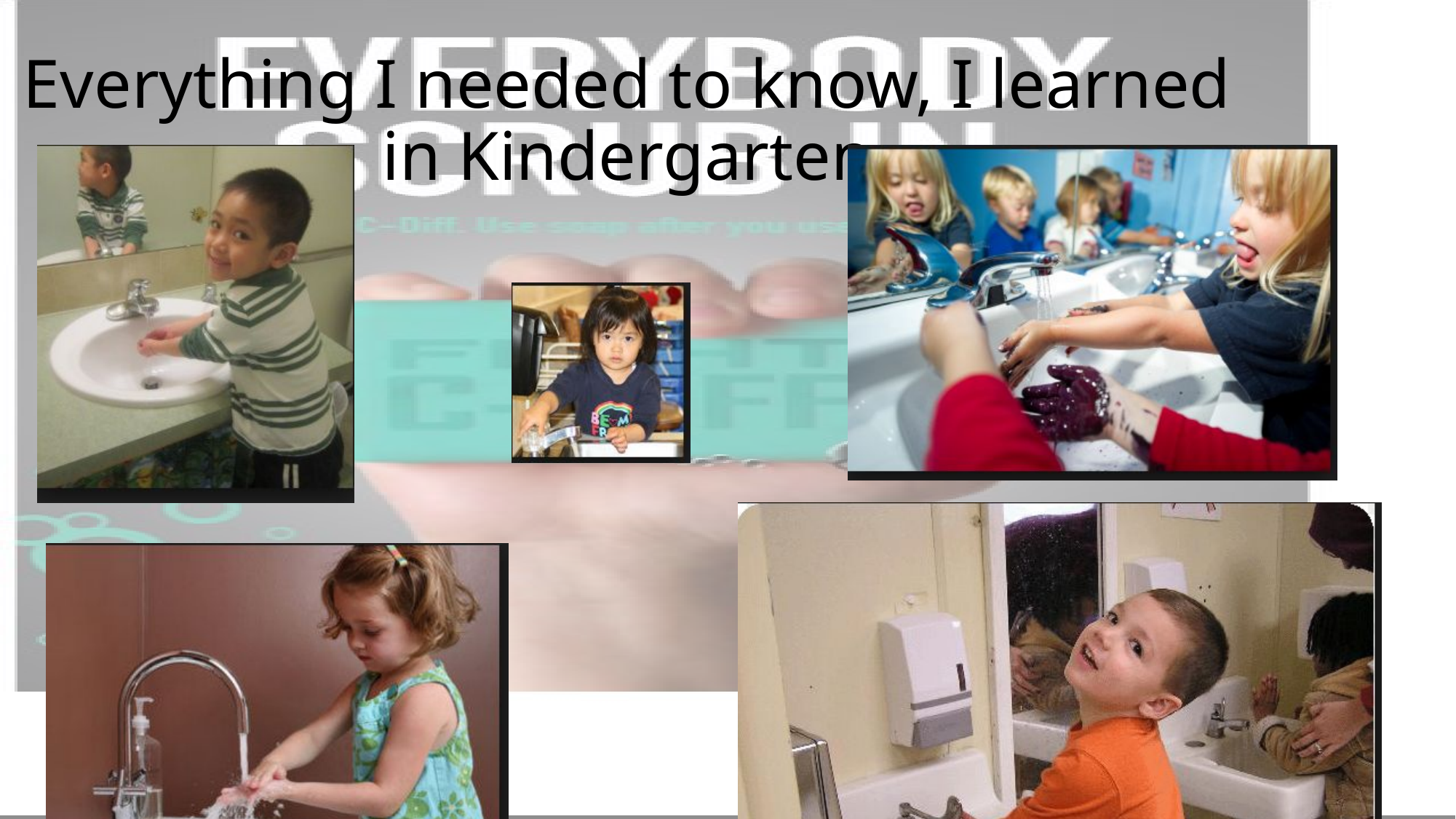

Everything I needed to know, I learned in Kindergarten

## Slide 3
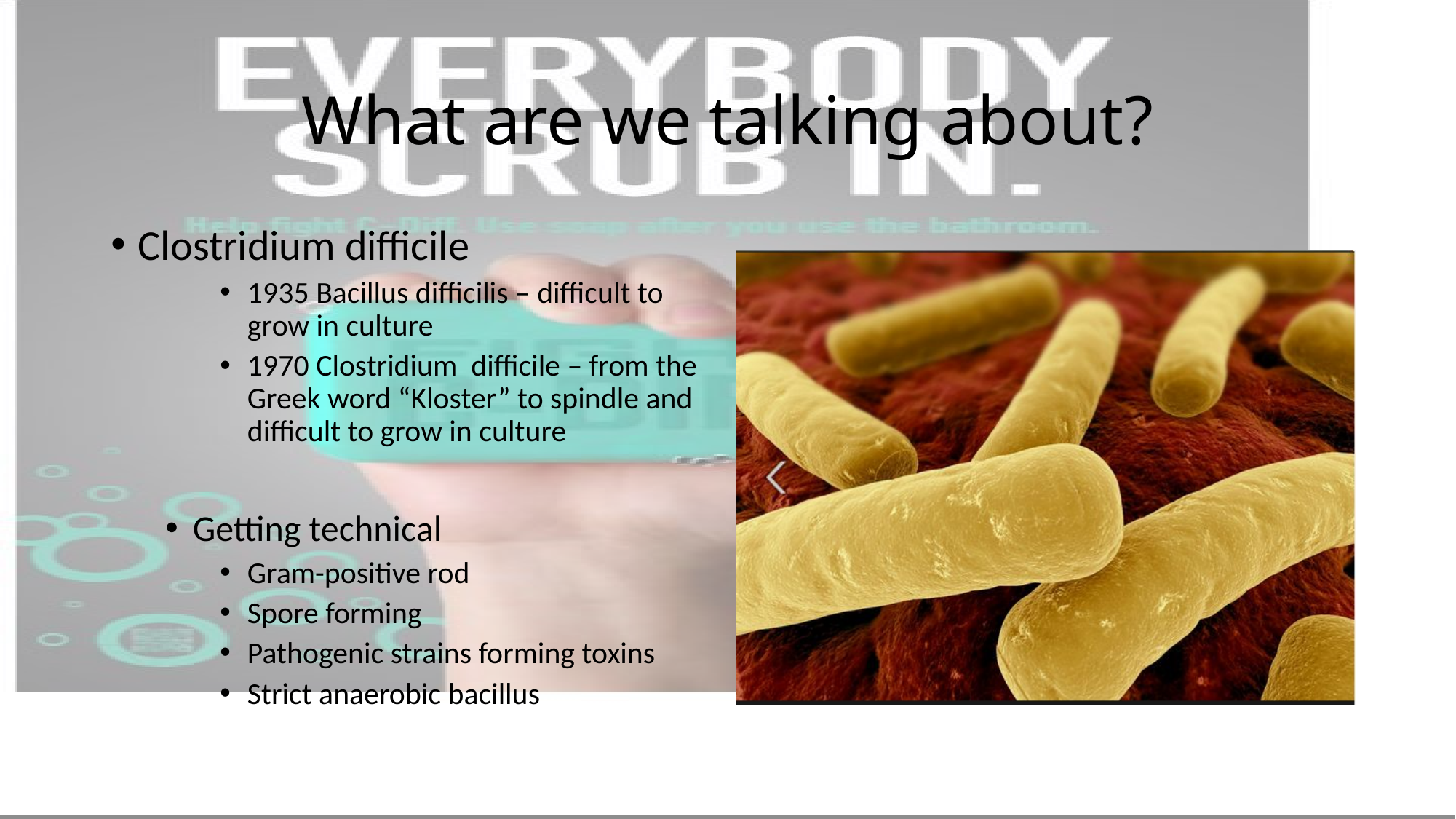

# What are we talking about?
Clostridium difficile
1935 Bacillus difficilis – difficult to grow in culture
1970 Clostridium difficile – from the Greek word “Kloster” to spindle and difficult to grow in culture
Getting technical
Gram-positive rod
Spore forming
Pathogenic strains forming toxins
Strict anaerobic bacillus

## Slide 4
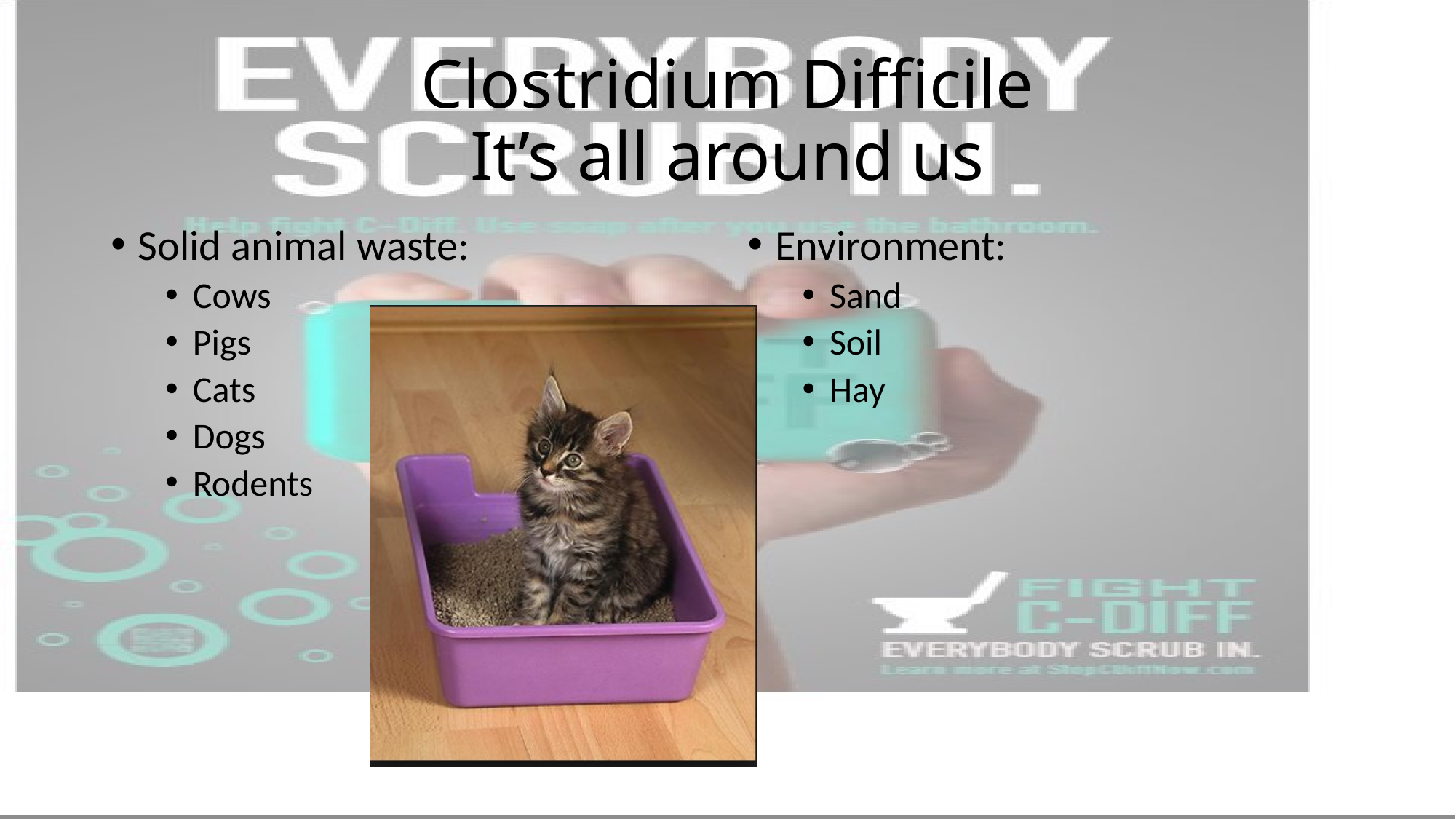

# Clostridium Difficile It’s all around us
Solid animal waste:
Cows
Pigs
Cats
Dogs
Rodents
Environment:
Sand
Soil
Hay

## Slide 5
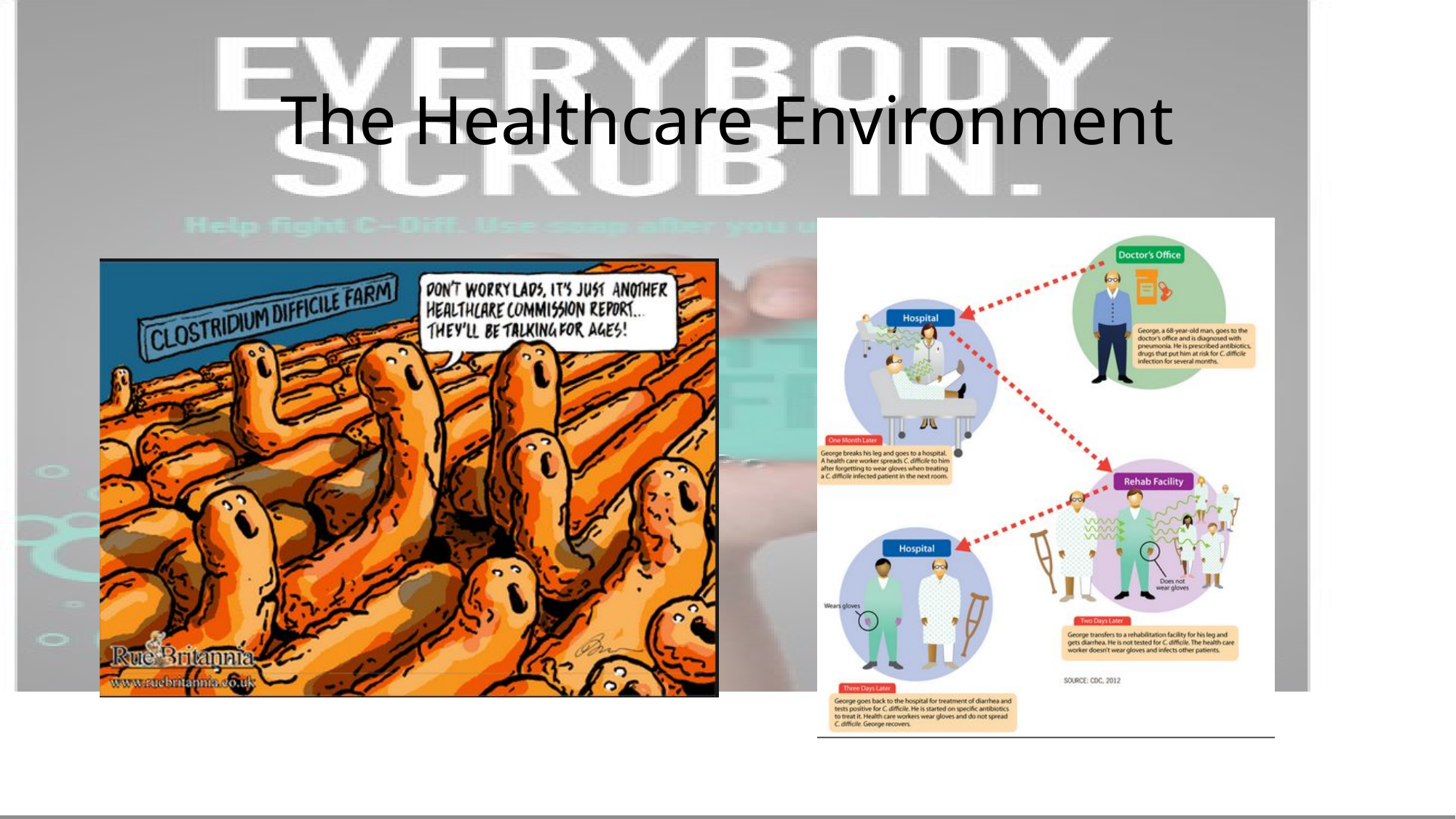

# The Healthcare Environment

## Slide 6
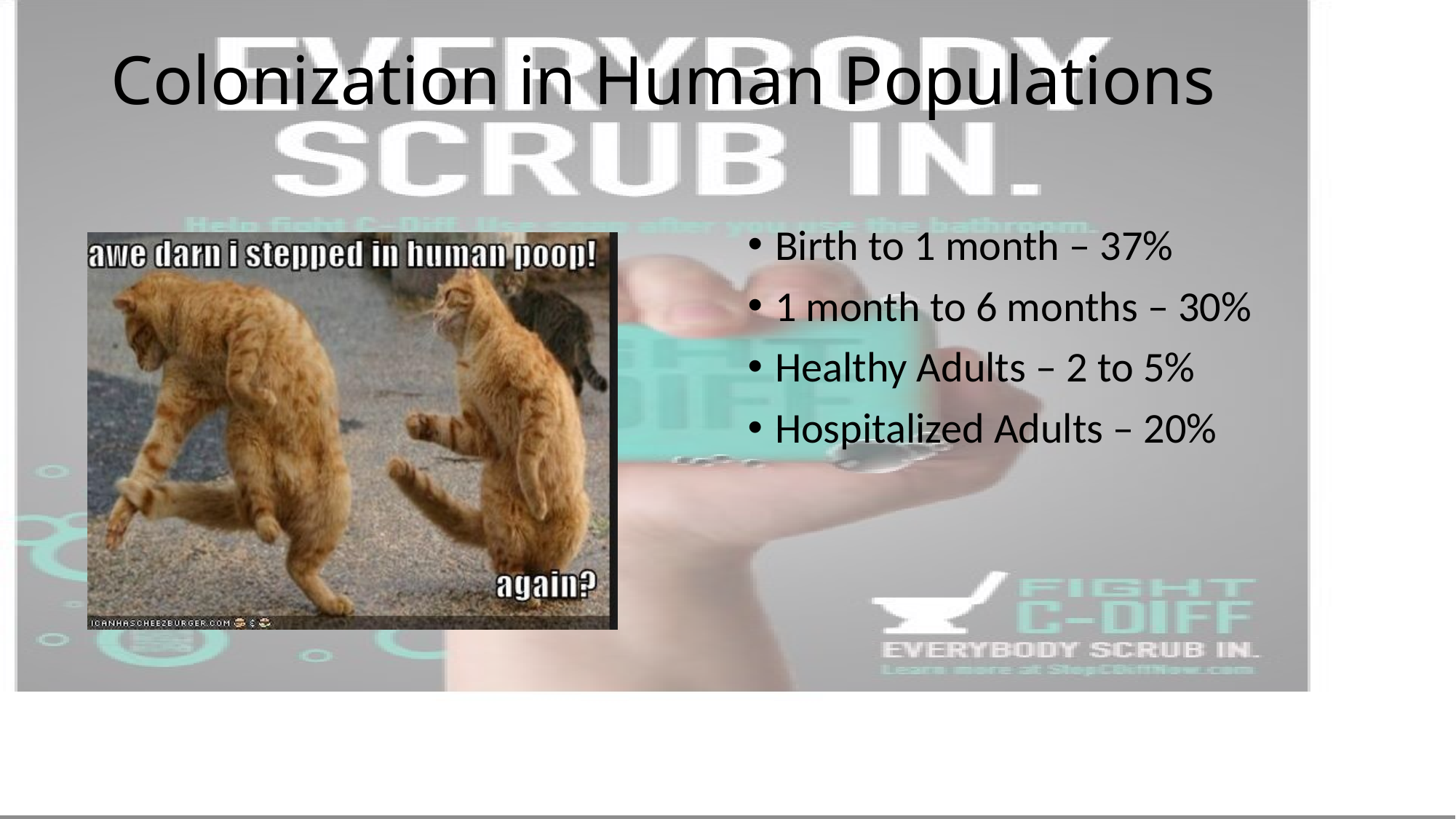

# Colonization in Human Populations
Birth to 1 month – 37%
1 month to 6 months – 30%
Healthy Adults – 2 to 5%
Hospitalized Adults – 20%

## Slide 7
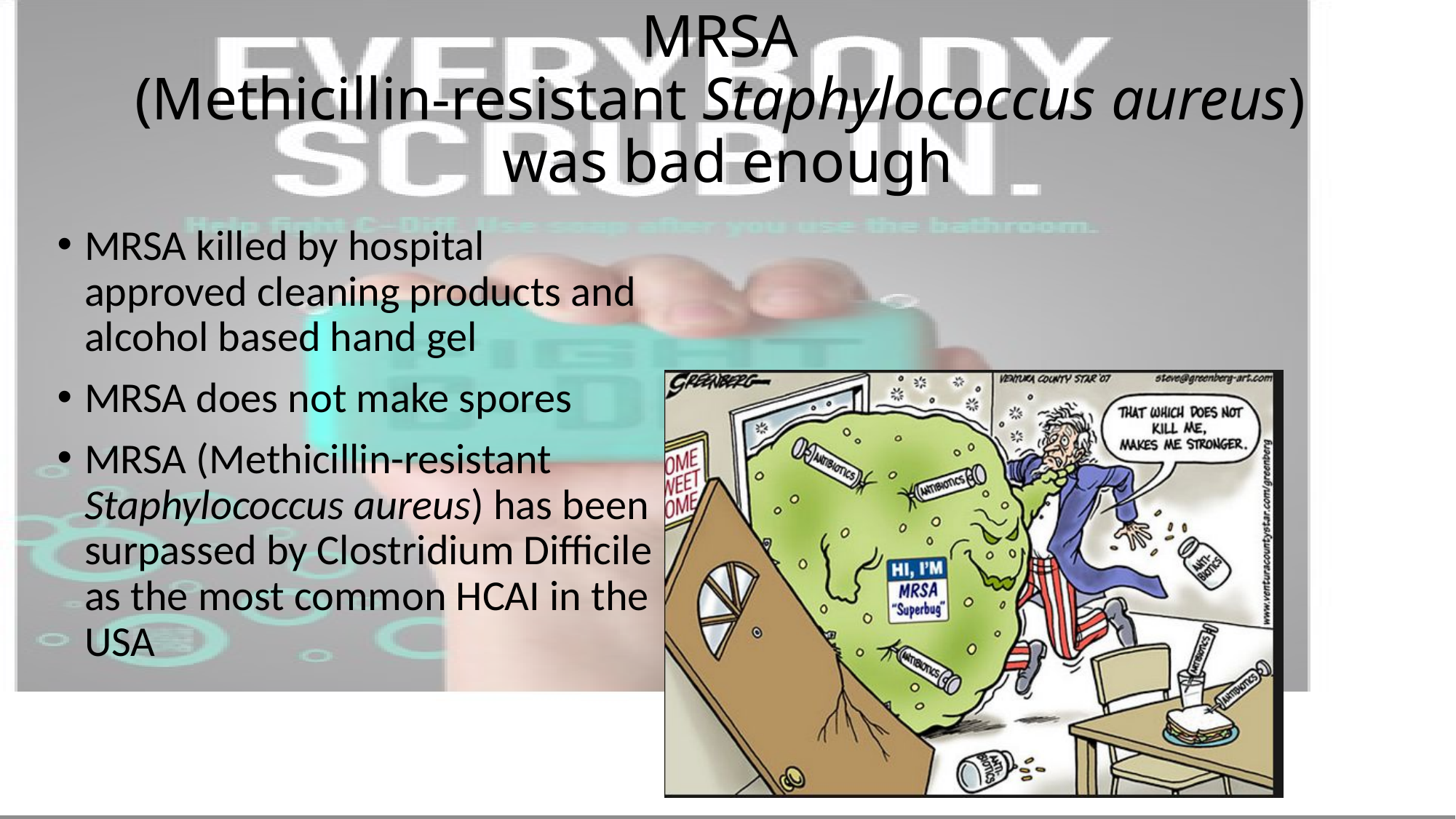

# MRSA (Methicillin-resistant Staphylococcus aureus) was bad enough
MRSA killed by hospital approved cleaning products and alcohol based hand gel
MRSA does not make spores
MRSA (Methicillin-resistant Staphylococcus aureus) has been surpassed by Clostridium Difficile as the most common HCAI in the USA

## Slide 8
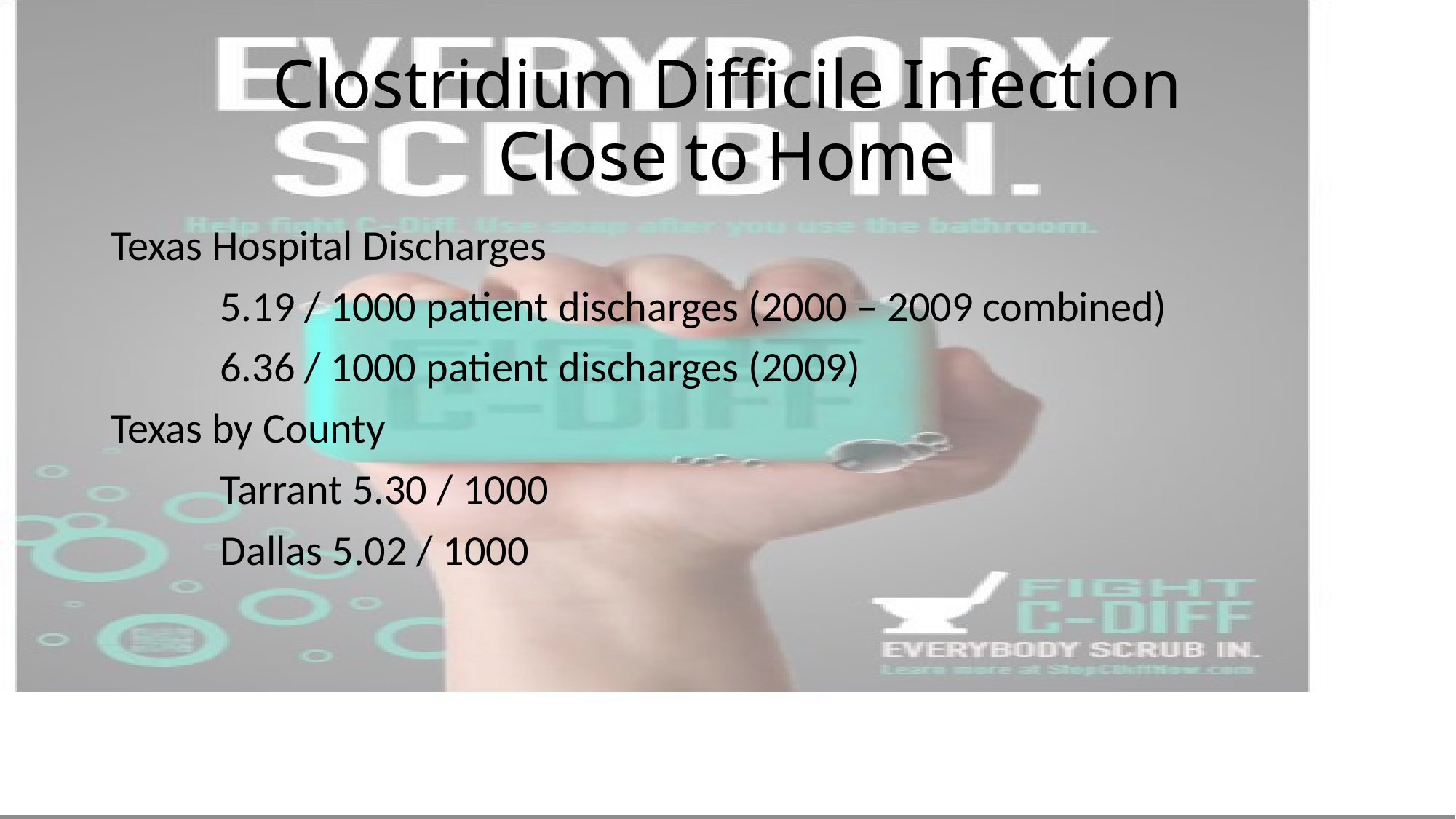

# Clostridium Difficile Infection Close to Home
Texas Hospital Discharges
	5.19 / 1000 patient discharges (2000 – 2009 combined)
	6.36 / 1000 patient discharges (2009)
Texas by County
	Tarrant 5.30 / 1000
	Dallas 5.02 / 1000

## Slide 9
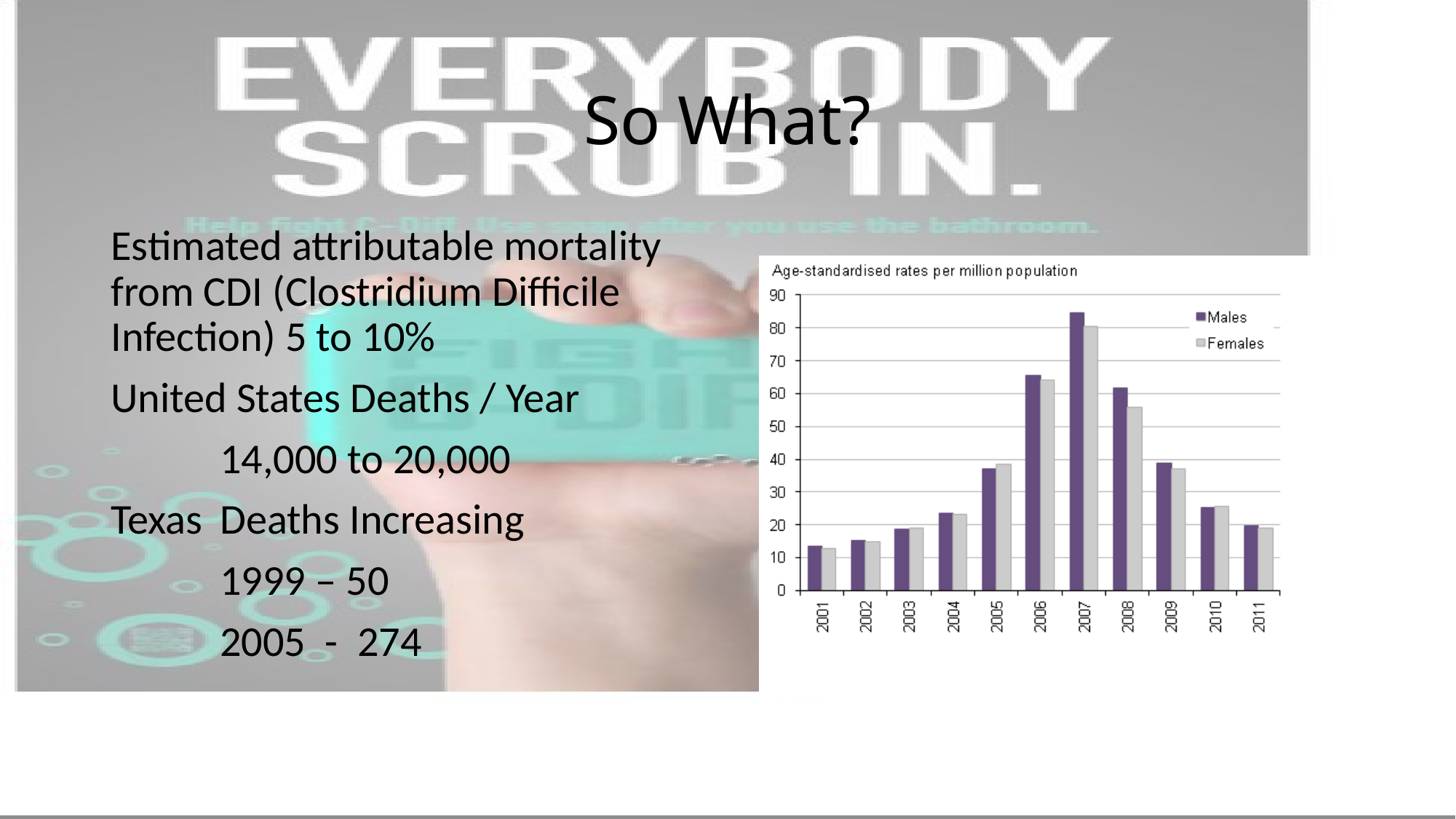

# So What?
Estimated attributable mortality from CDI (Clostridium Difficile Infection) 5 to 10%
United States Deaths / Year
	14,000 to 20,000
Texas	Deaths Increasing
	1999 – 50
	2005 - 274

## Slide 10
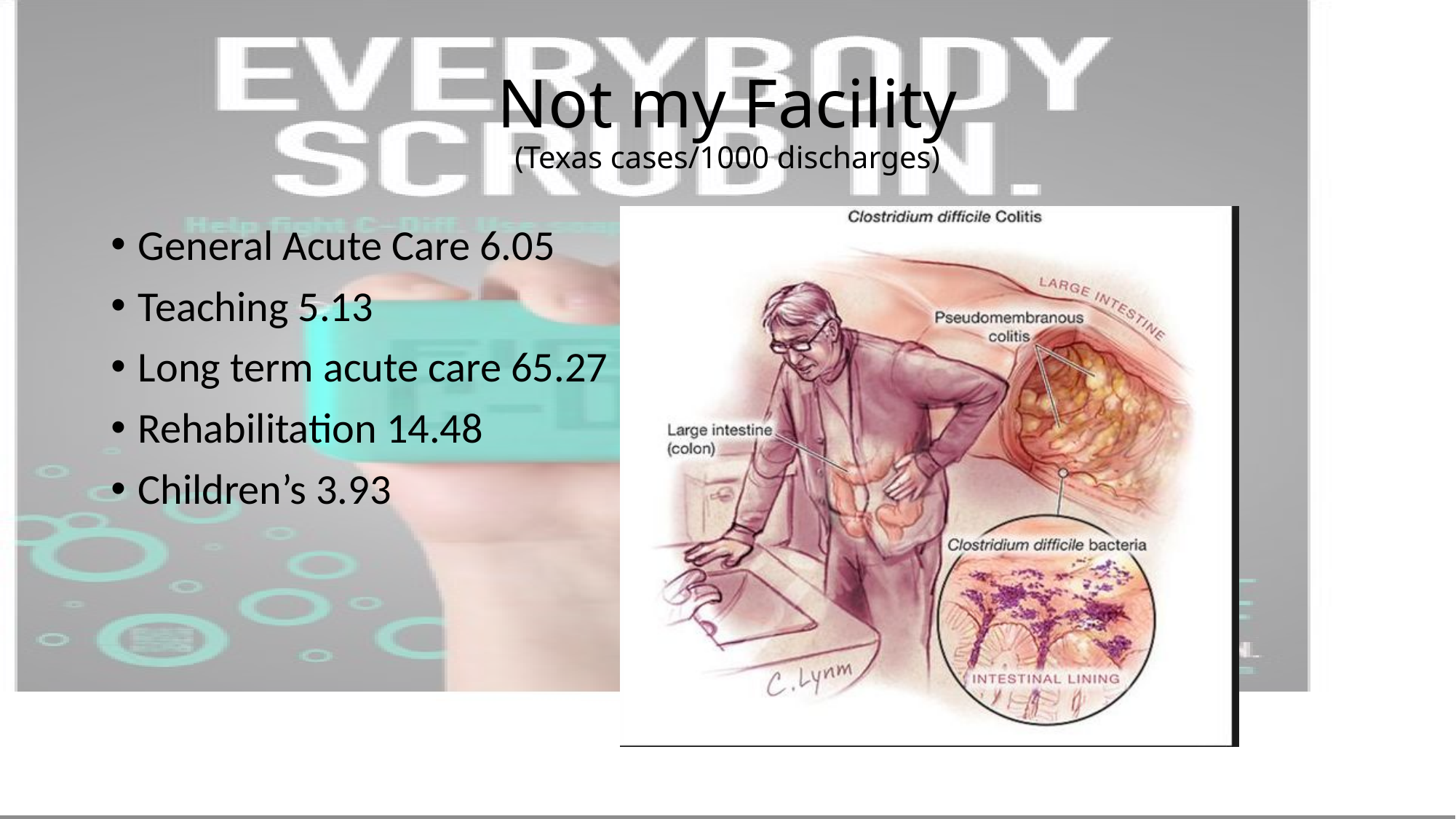

# Not my Facility (Texas cases/1000 discharges)
General Acute Care 6.05
Teaching 5.13
Long term acute care 65.27
Rehabilitation 14.48
Children’s 3.93

## Slide 11
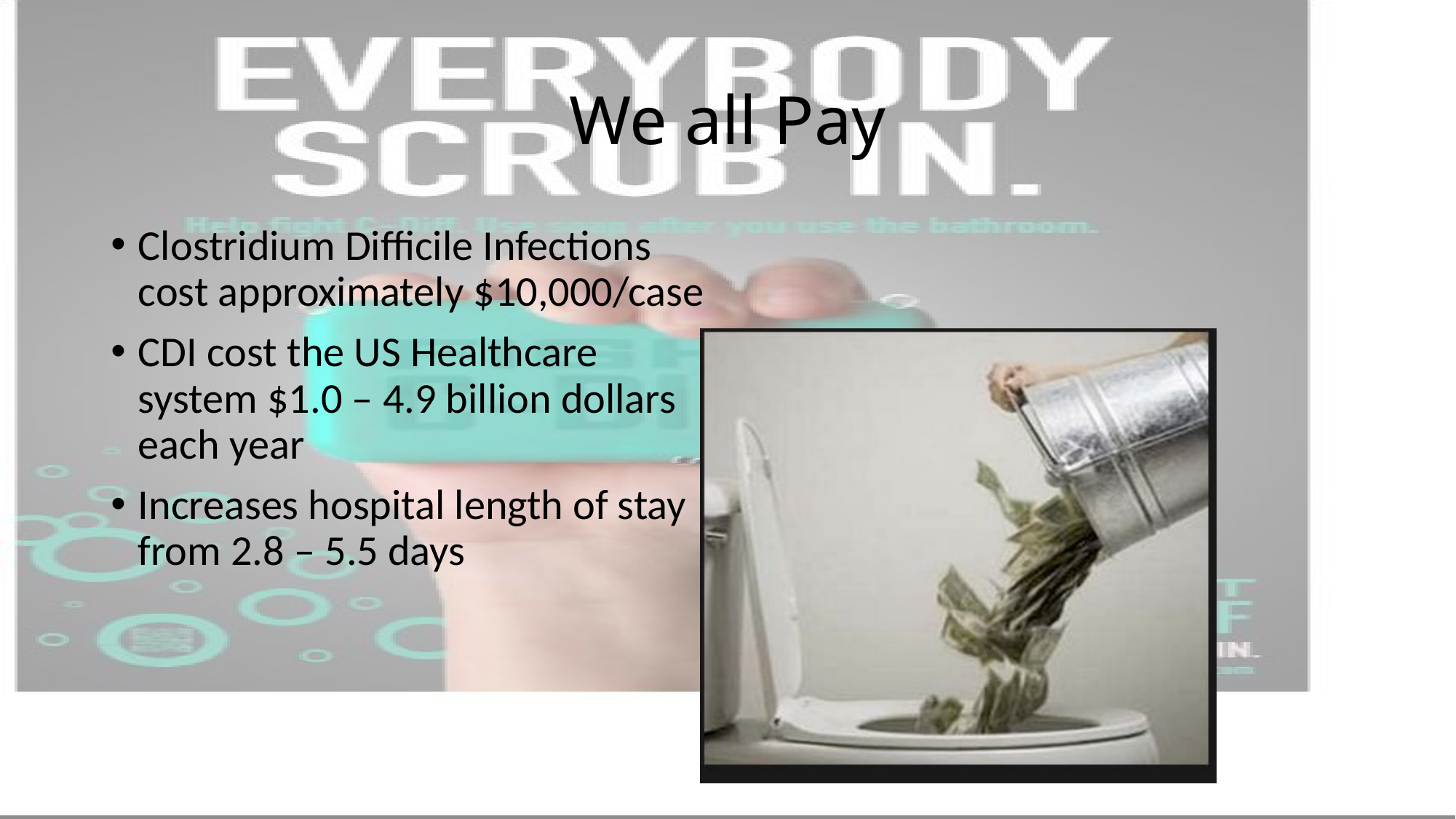

# We all Pay
Clostridium Difficile Infections cost approximately $10,000/case
CDI cost the US Healthcare system $1.0 – 4.9 billion dollars each year
Increases hospital length of stay from 2.8 – 5.5 days

## Slide 12
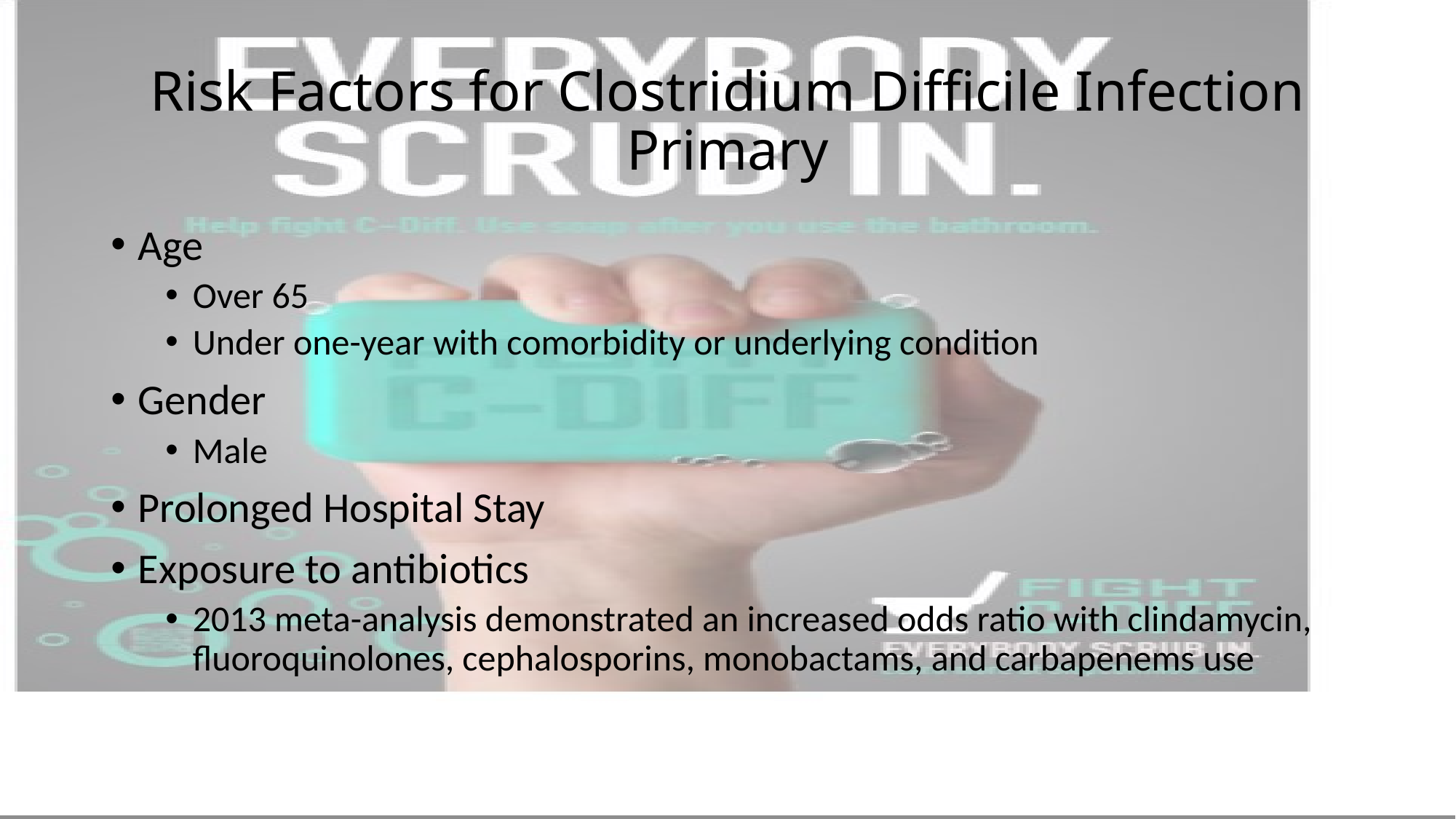

# Risk Factors for Clostridium Difficile Infection Primary
Age
Over 65
Under one-year with comorbidity or underlying condition
Gender
Male
Prolonged Hospital Stay
Exposure to antibiotics
2013 meta-analysis demonstrated an increased odds ratio with clindamycin, fluoroquinolones, cephalosporins, monobactams, and carbapenems use

## Slide 13
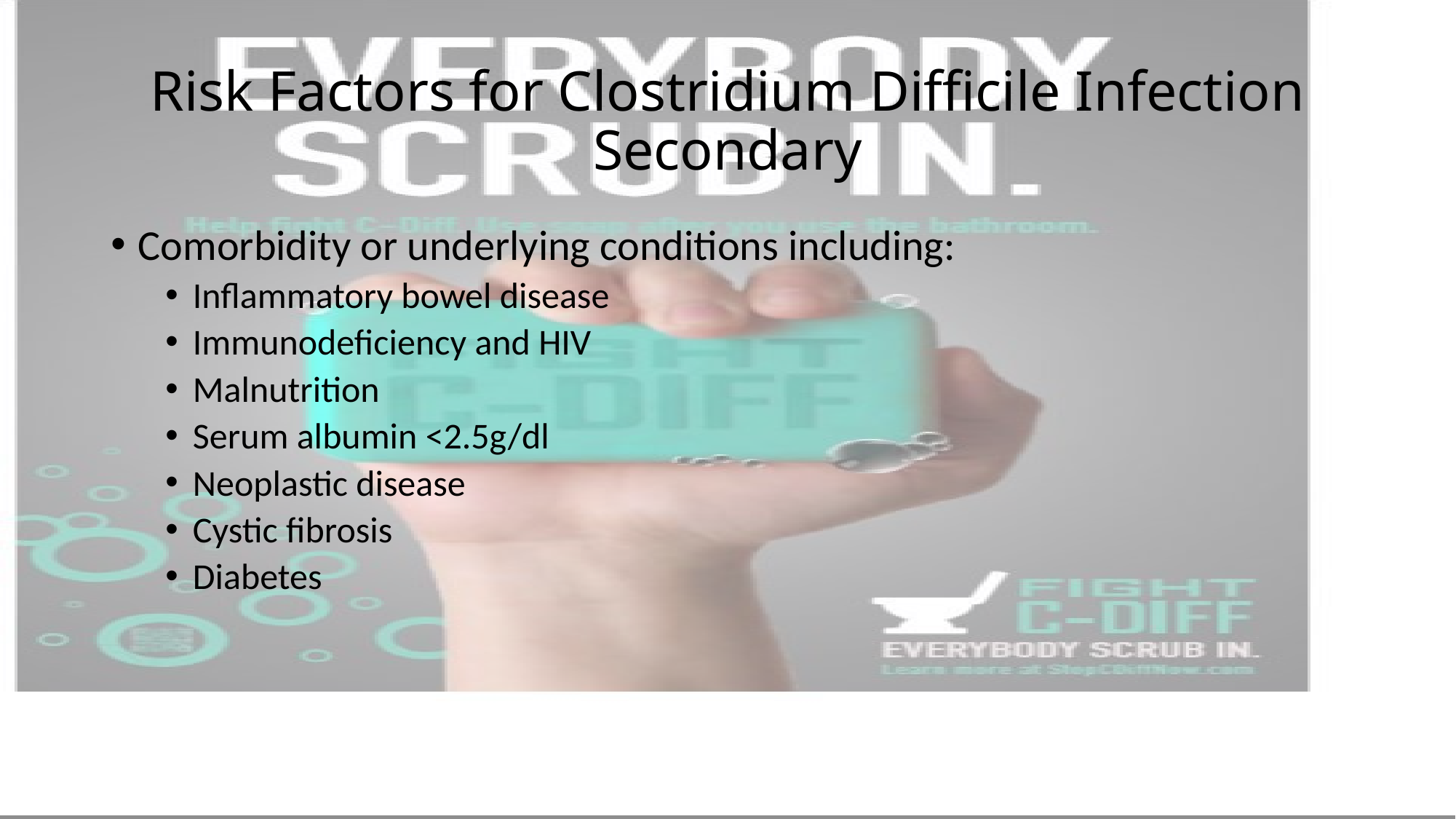

# Risk Factors for Clostridium Difficile Infection Secondary
Comorbidity or underlying conditions including:
Inflammatory bowel disease
Immunodeficiency and HIV
Malnutrition
Serum albumin <2.5g/dl
Neoplastic disease
Cystic fibrosis
Diabetes

## Slide 14
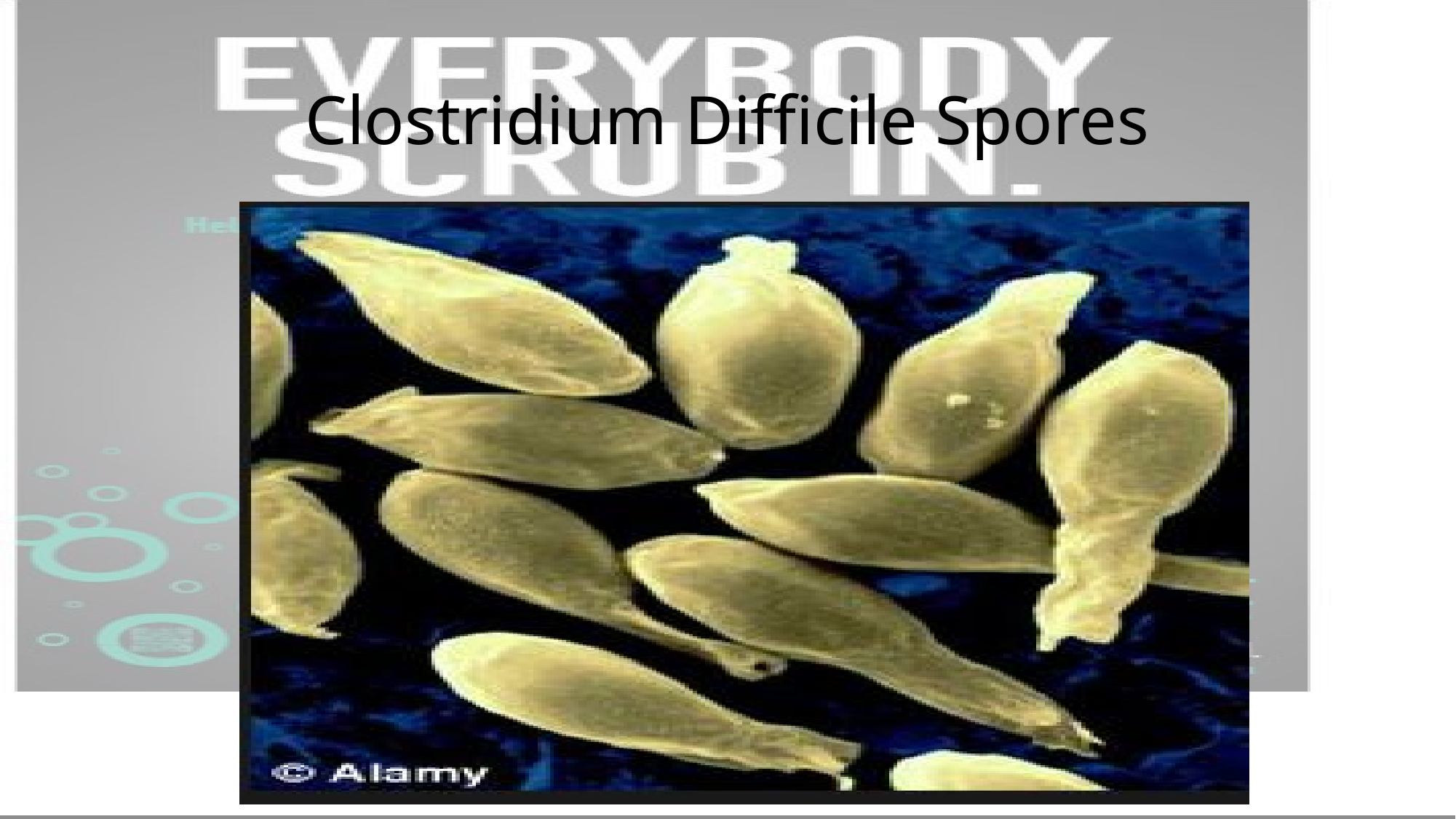

# Clostridium Difficile Spores

## Slide 15
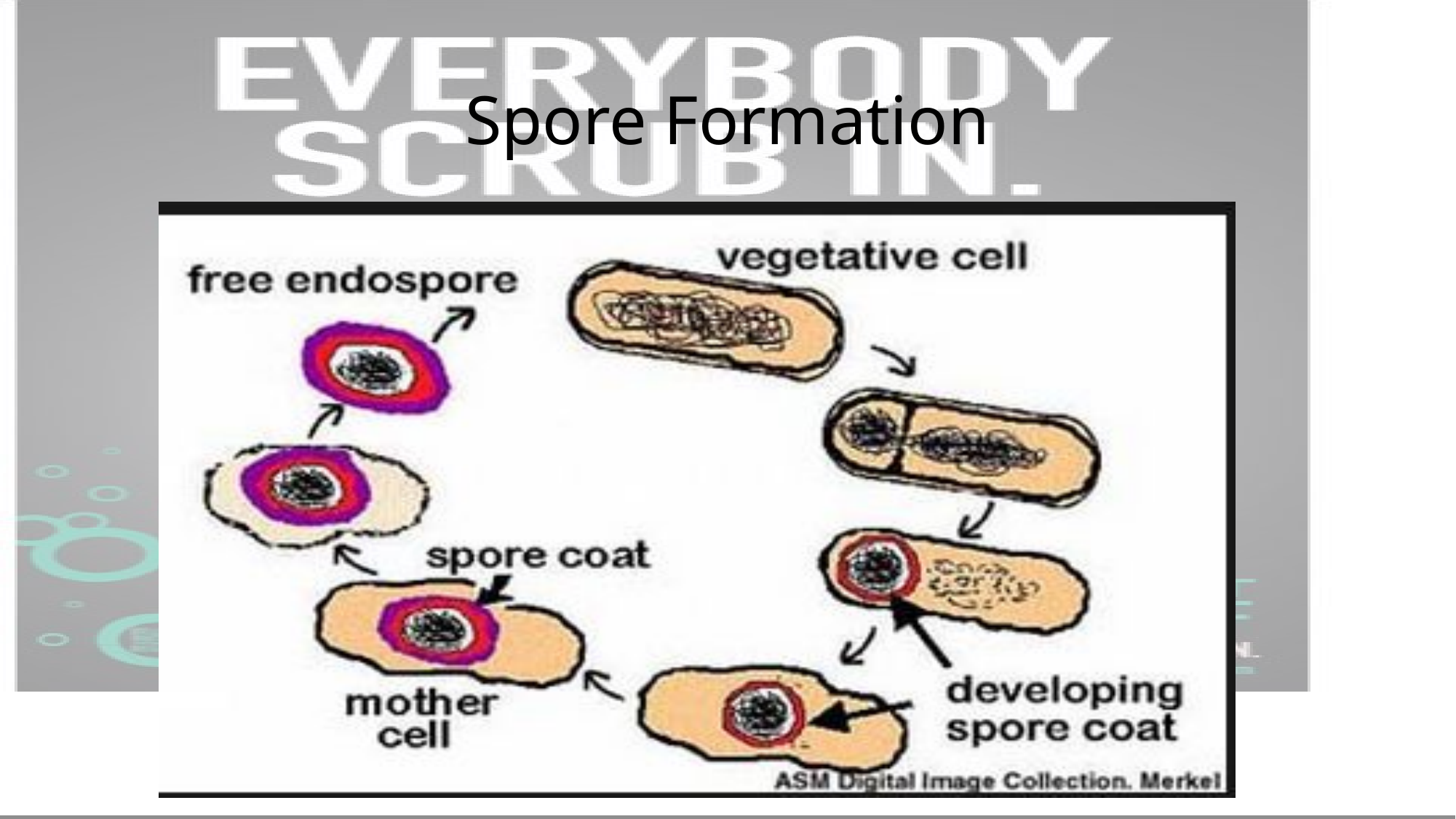

# Spore Formation

## Slide 16
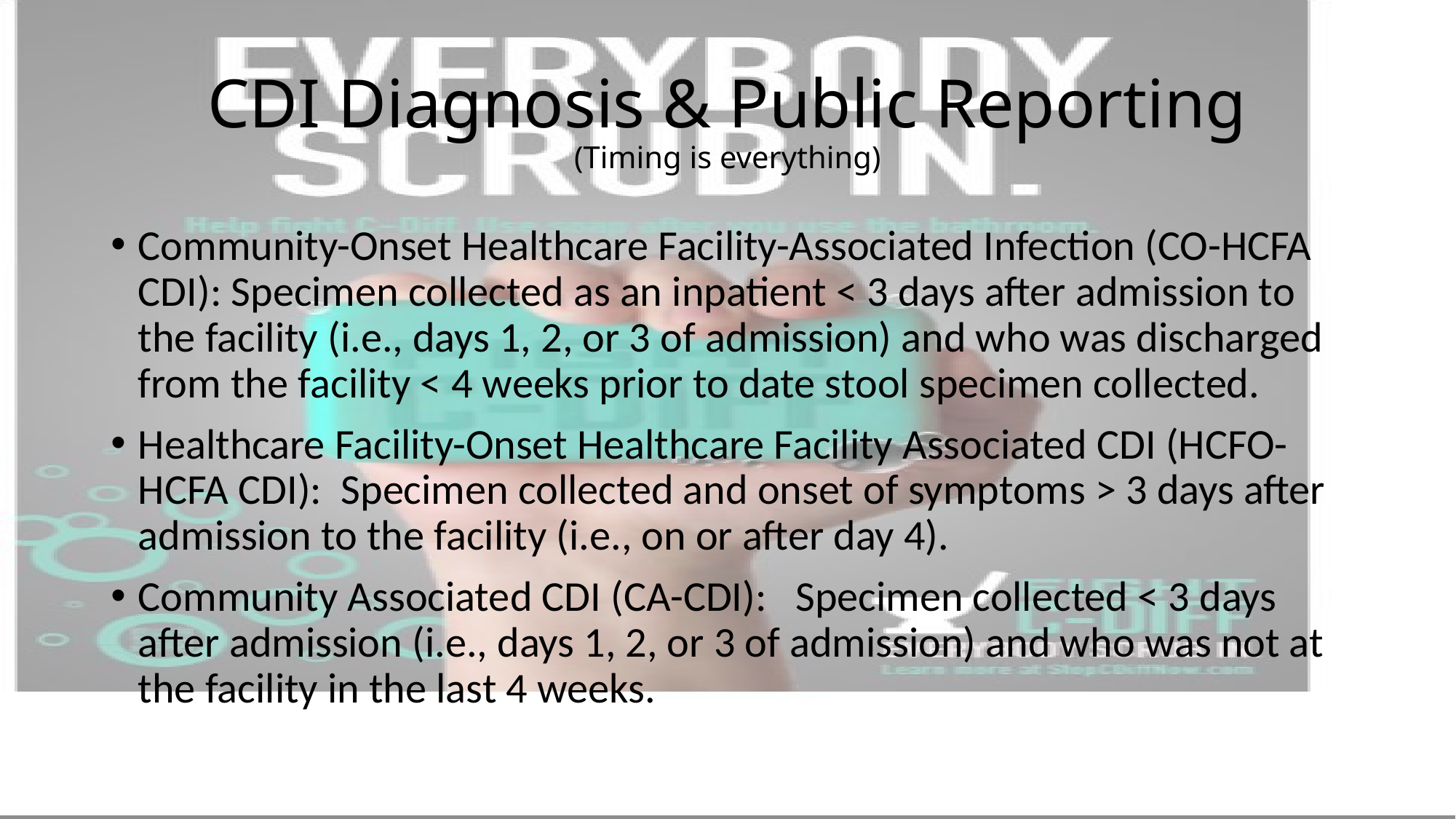

# CDI Diagnosis & Public Reporting (Timing is everything)
Community-Onset Healthcare Facility-Associated Infection (CO-HCFA CDI): Specimen collected as an inpatient < 3 days after admission to the facility (i.e., days 1, 2, or 3 of admission) and who was discharged from the facility < 4 weeks prior to date stool specimen collected.
Healthcare Facility-Onset Healthcare Facility Associated CDI (HCFO-HCFA CDI): Specimen collected and onset of symptoms > 3 days after admission to the facility (i.e., on or after day 4).
Community Associated CDI (CA-CDI): Specimen collected < 3 days after admission (i.e., days 1, 2, or 3 of admission) and who was not at the facility in the last 4 weeks.

## Slide 17
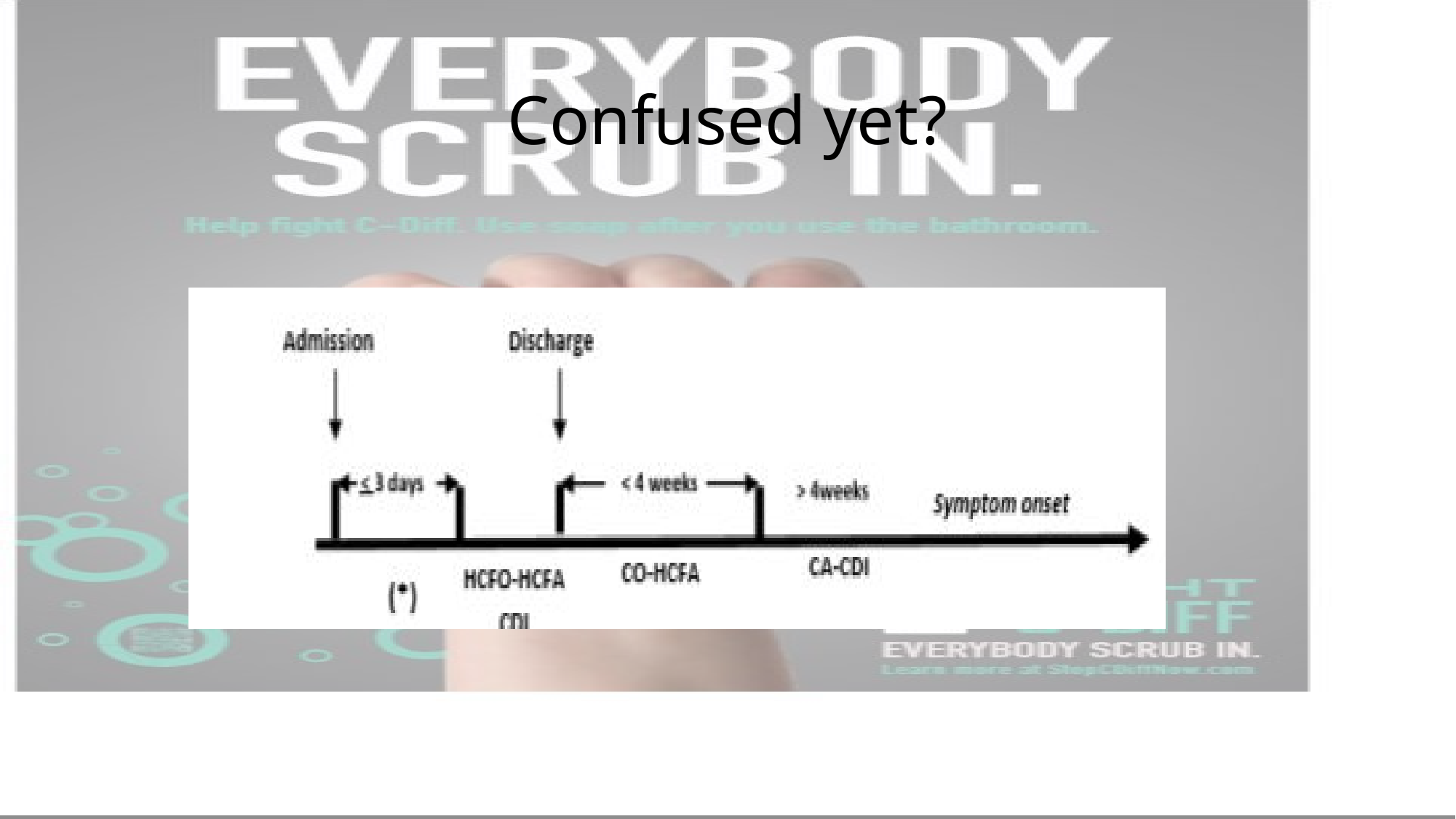

# Confused yet?

## Slide 18
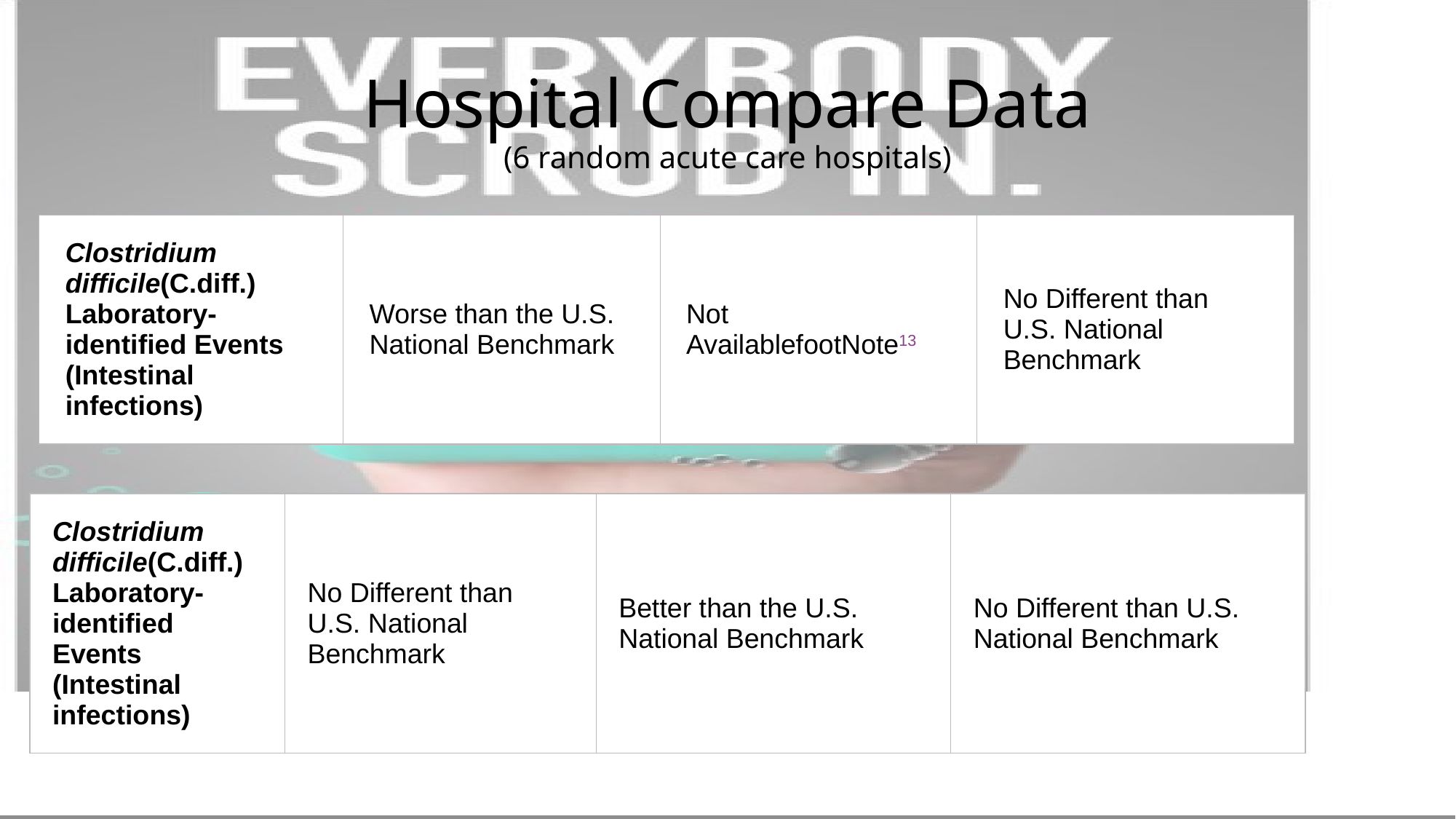

# Hospital Compare Data (6 random acute care hospitals)
| Clostridium difficile(C.diff.) Laboratory-identified Events (Intestinal infections) | Worse than the U.S. National Benchmark | Not AvailablefootNote13 | No Different than U.S. National Benchmark |
| --- | --- | --- | --- |
| Clostridium difficile(C.diff.) Laboratory-identified Events (Intestinal infections) | No Different than U.S. National Benchmark | Better than the U.S. National Benchmark | No Different than U.S. National Benchmark |
| --- | --- | --- | --- |

## Slide 19
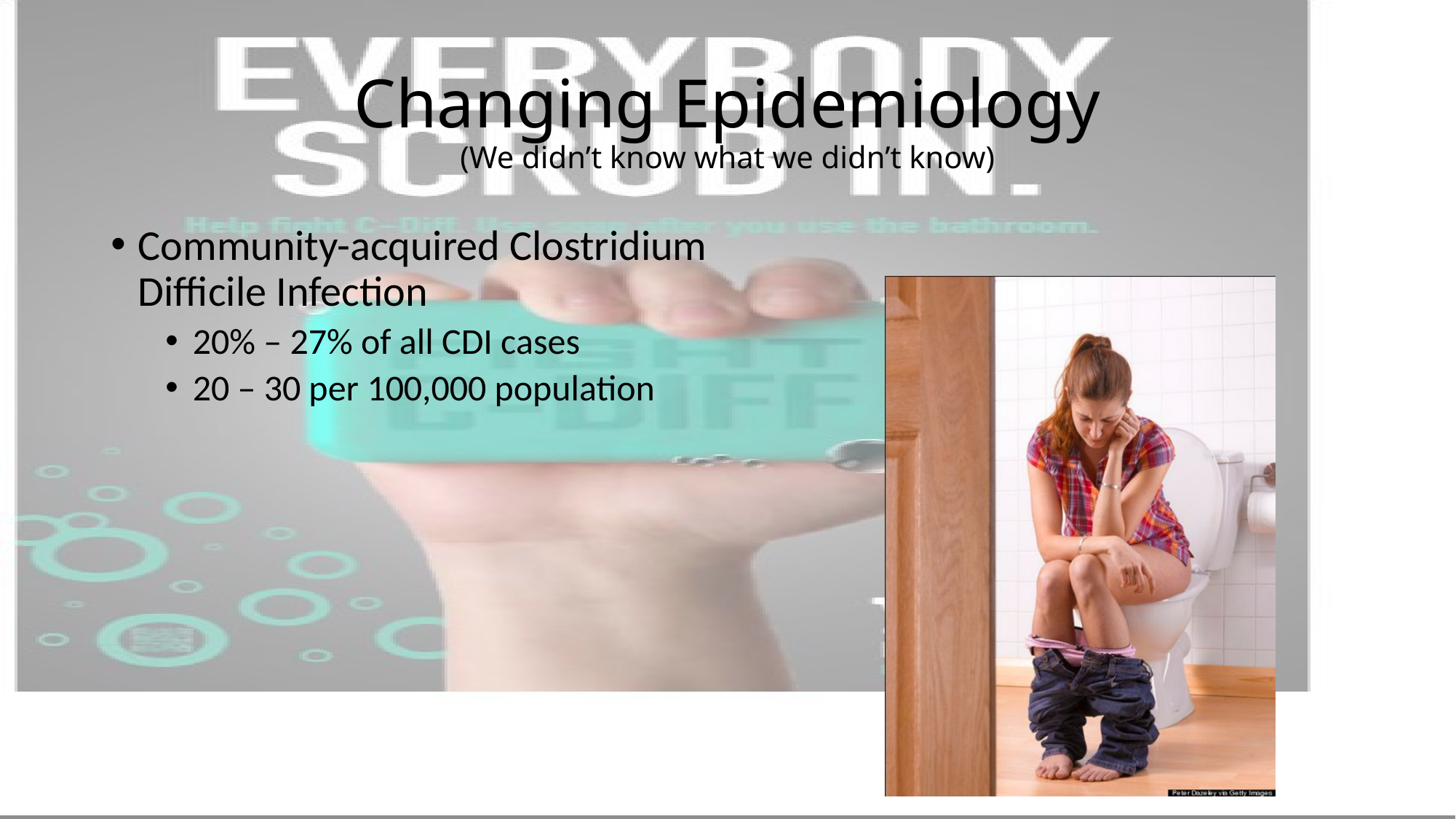

# Changing Epidemiology (We didn’t know what we didn’t know)
Community-acquired Clostridium Difficile Infection
20% – 27% of all CDI cases
20 – 30 per 100,000 population

## Slide 20
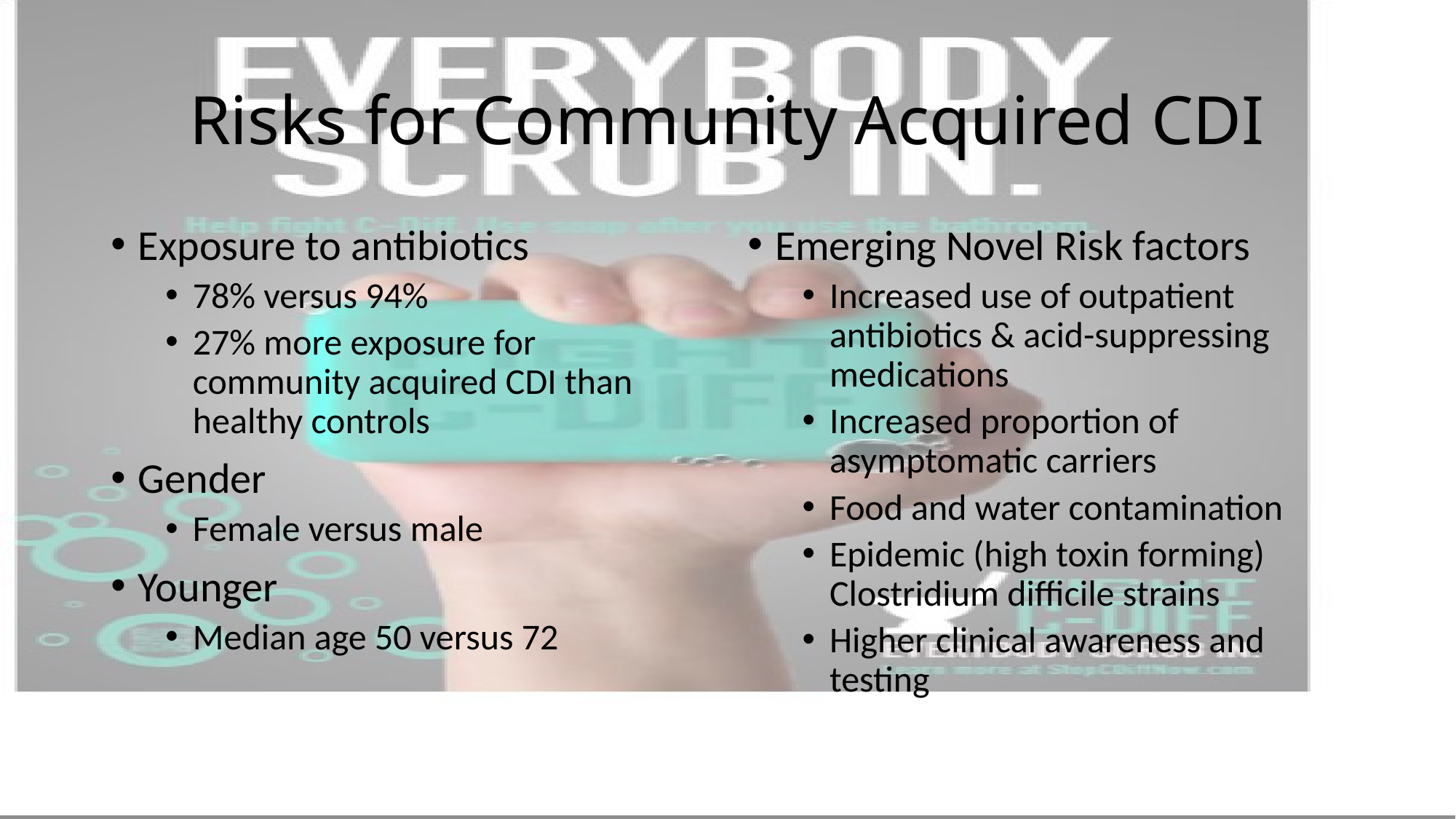

# Risks for Community Acquired CDI
Exposure to antibiotics
78% versus 94%
27% more exposure for community acquired CDI than healthy controls
Gender
Female versus male
Younger
Median age 50 versus 72
Emerging Novel Risk factors
Increased use of outpatient antibiotics & acid-suppressing medications
Increased proportion of asymptomatic carriers
Food and water contamination
Epidemic (high toxin forming) Clostridium difficile strains
Higher clinical awareness and testing

## Slide 21
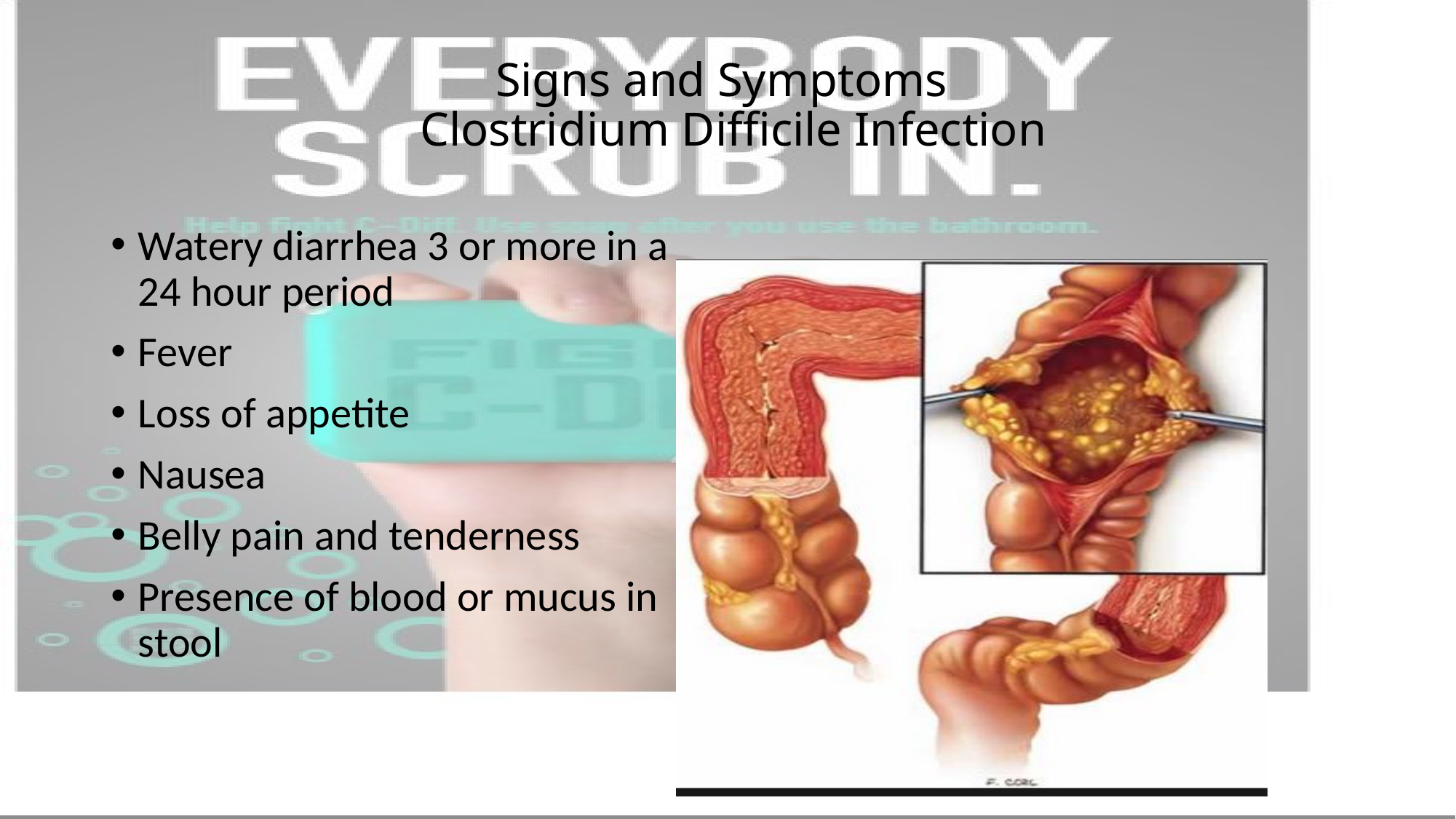

# Signs and Symptoms Clostridium Difficile Infection
Watery diarrhea 3 or more in a 24 hour period
Fever
Loss of appetite
Nausea
Belly pain and tenderness
Presence of blood or mucus in stool

## Slide 22
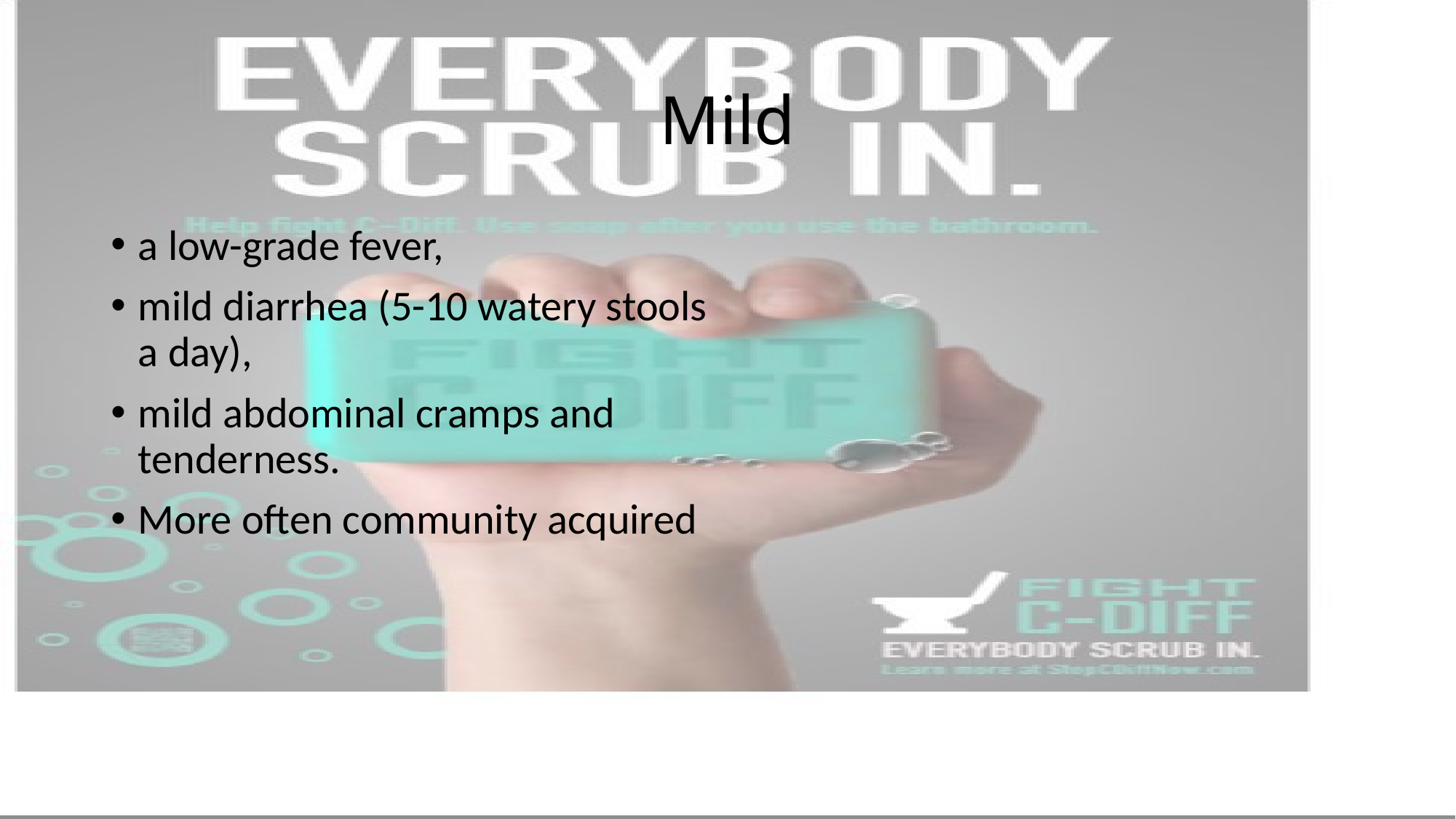

# Mild
a low-grade fever,
mild diarrhea (5-10 watery stools a day),
mild abdominal cramps and tenderness.
More often community acquired

## Slide 23
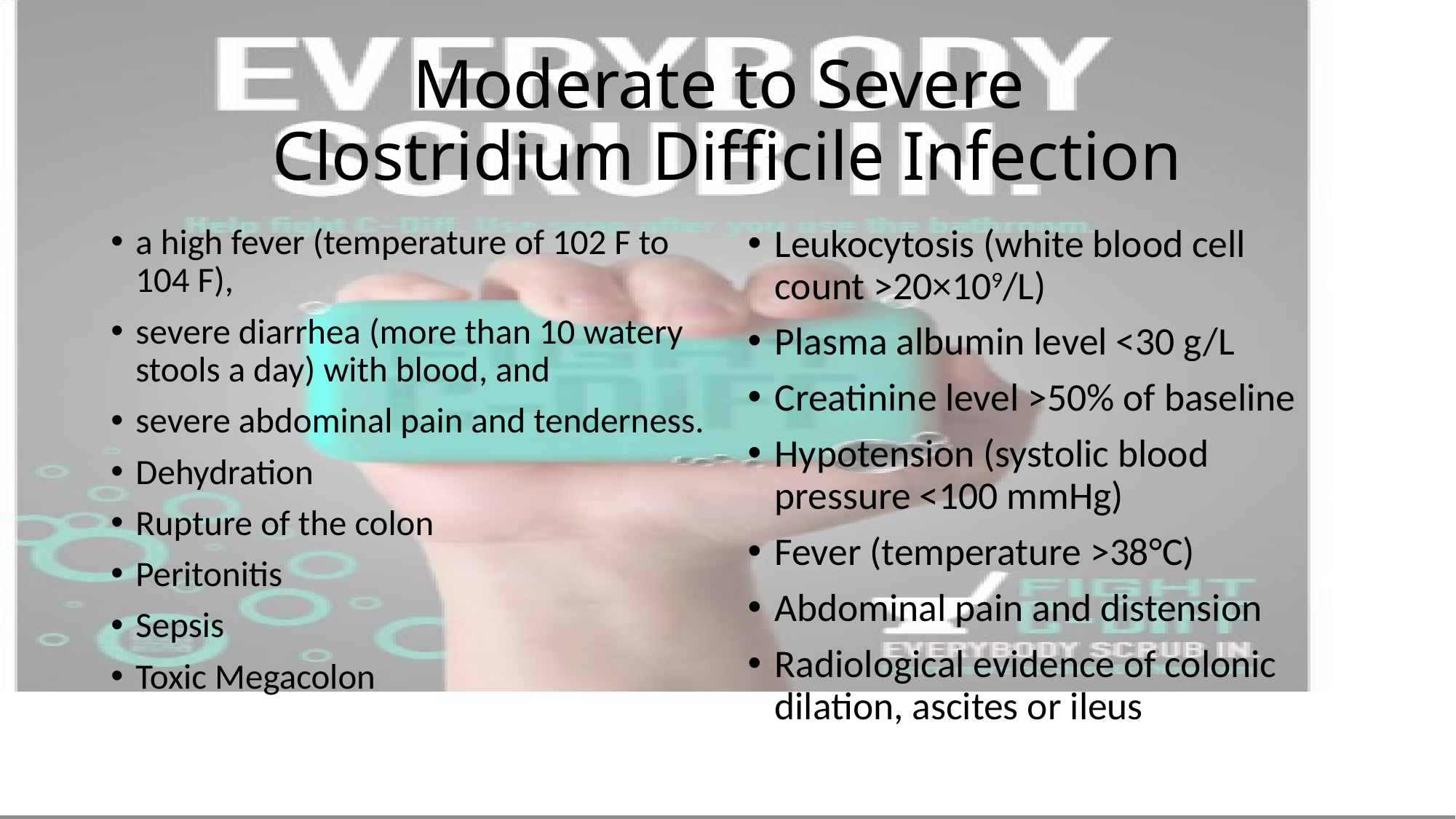

# Moderate to Severe Clostridium Difficile Infection
a high fever (temperature of 102 F to 104 F),
severe diarrhea (more than 10 watery stools a day) with blood, and
severe abdominal pain and tenderness.
Dehydration
Rupture of the colon
Peritonitis
Sepsis
Toxic Megacolon
Leukocytosis (white blood cell count >20×109/L)
Plasma albumin level <30 g/L
Creatinine level >50% of baseline
Hypotension (systolic blood pressure <100 mmHg)
Fever (temperature >38°C)
Abdominal pain and distension
Radiological evidence of colonic dilation, ascites or ileus

## Slide 24
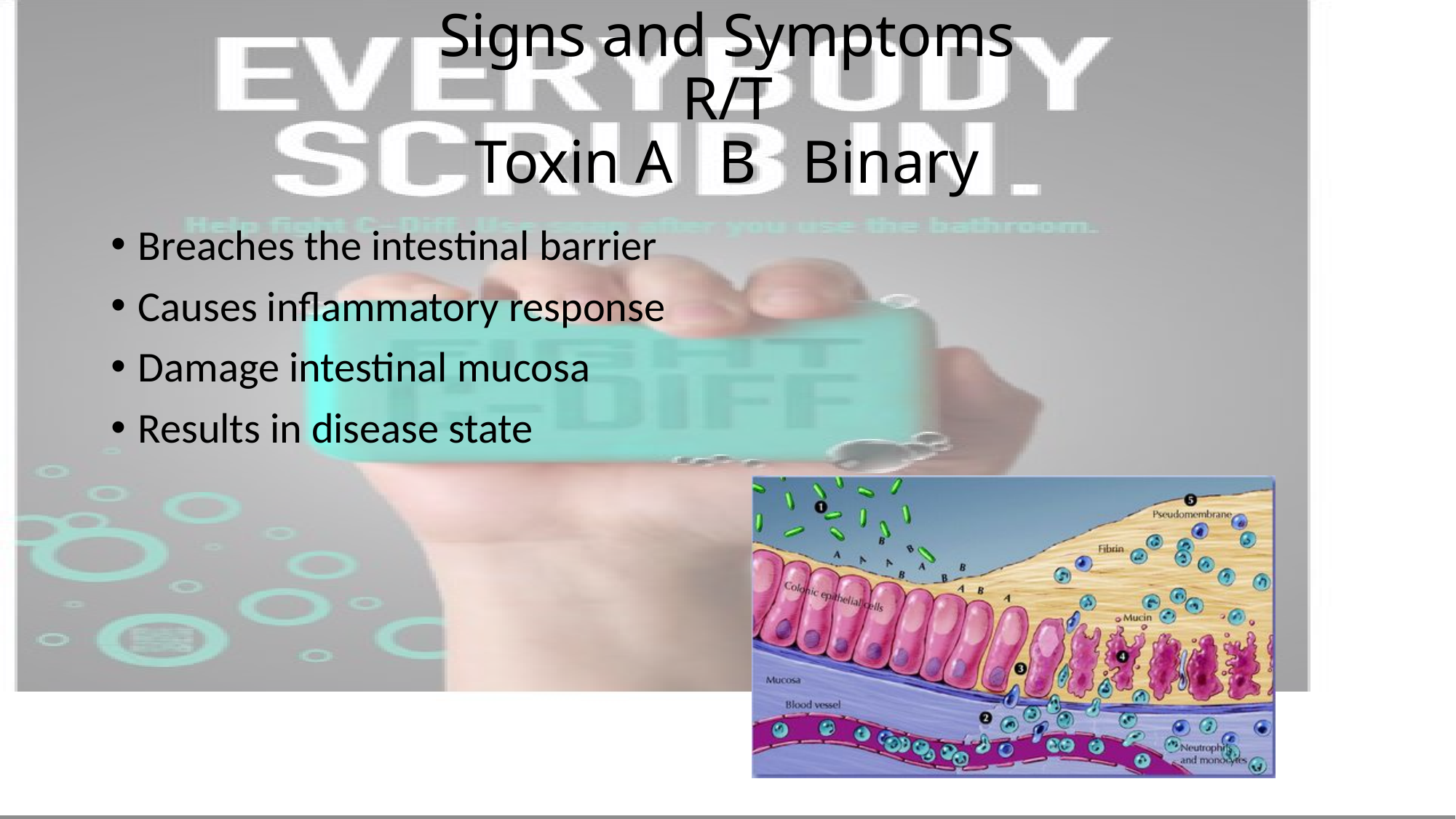

# Signs and Symptoms R/T Toxin A B Binary
Breaches the intestinal barrier
Causes inflammatory response
Damage intestinal mucosa
Results in disease state

## Slide 25
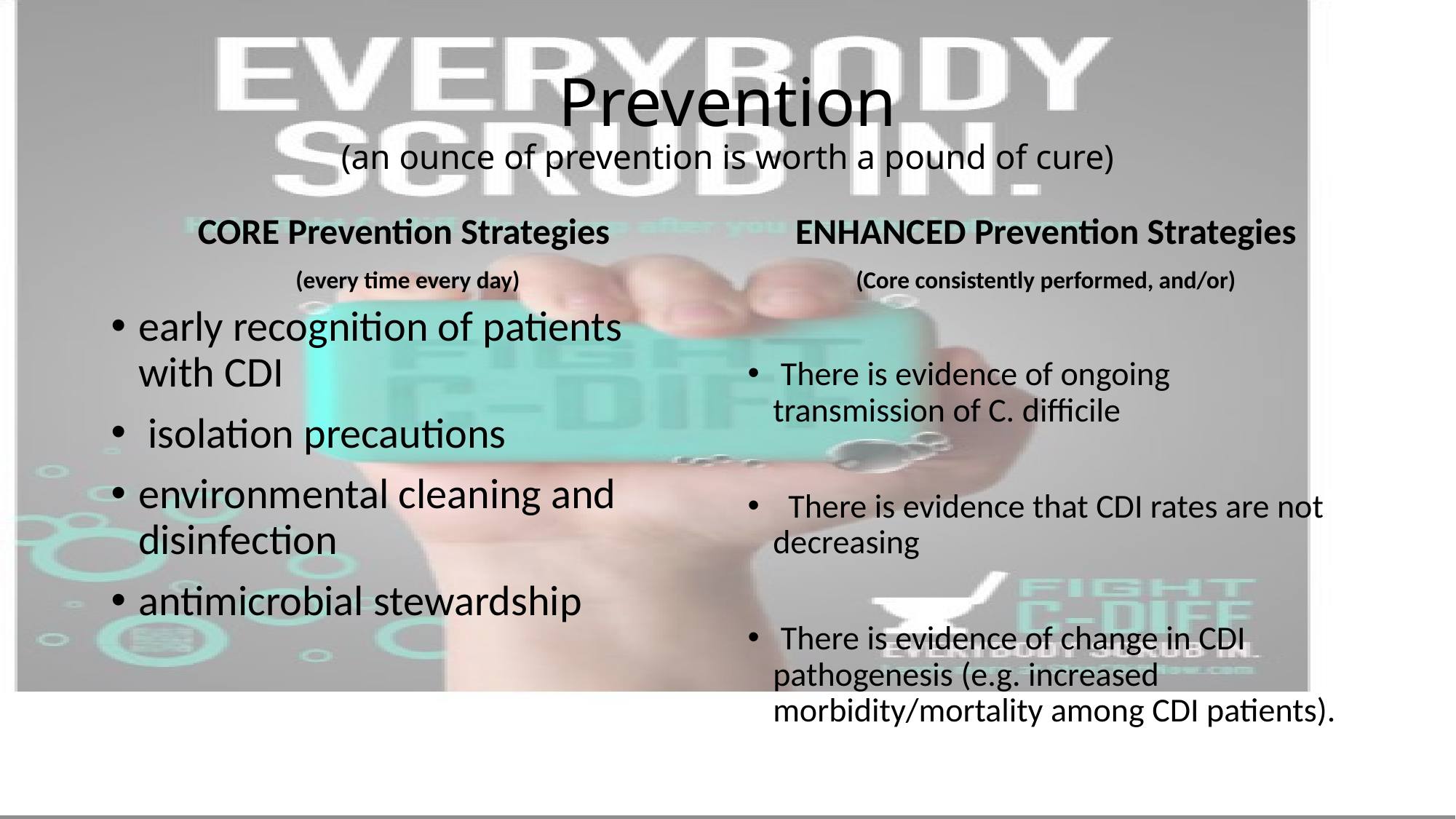

# Prevention (an ounce of prevention is worth a pound of cure)
CORE Prevention Strategies
(every time every day)
ENHANCED Prevention Strategies
(Core consistently performed, and/or)
early recognition of patients with CDI
 isolation precautions
environmental cleaning and disinfection
antimicrobial stewardship
 There is evidence of ongoing transmission of C. difficile
 There is evidence that CDI rates are not decreasing
 There is evidence of change in CDI pathogenesis (e.g. increased morbidity/mortality among CDI patients).

## Slide 26
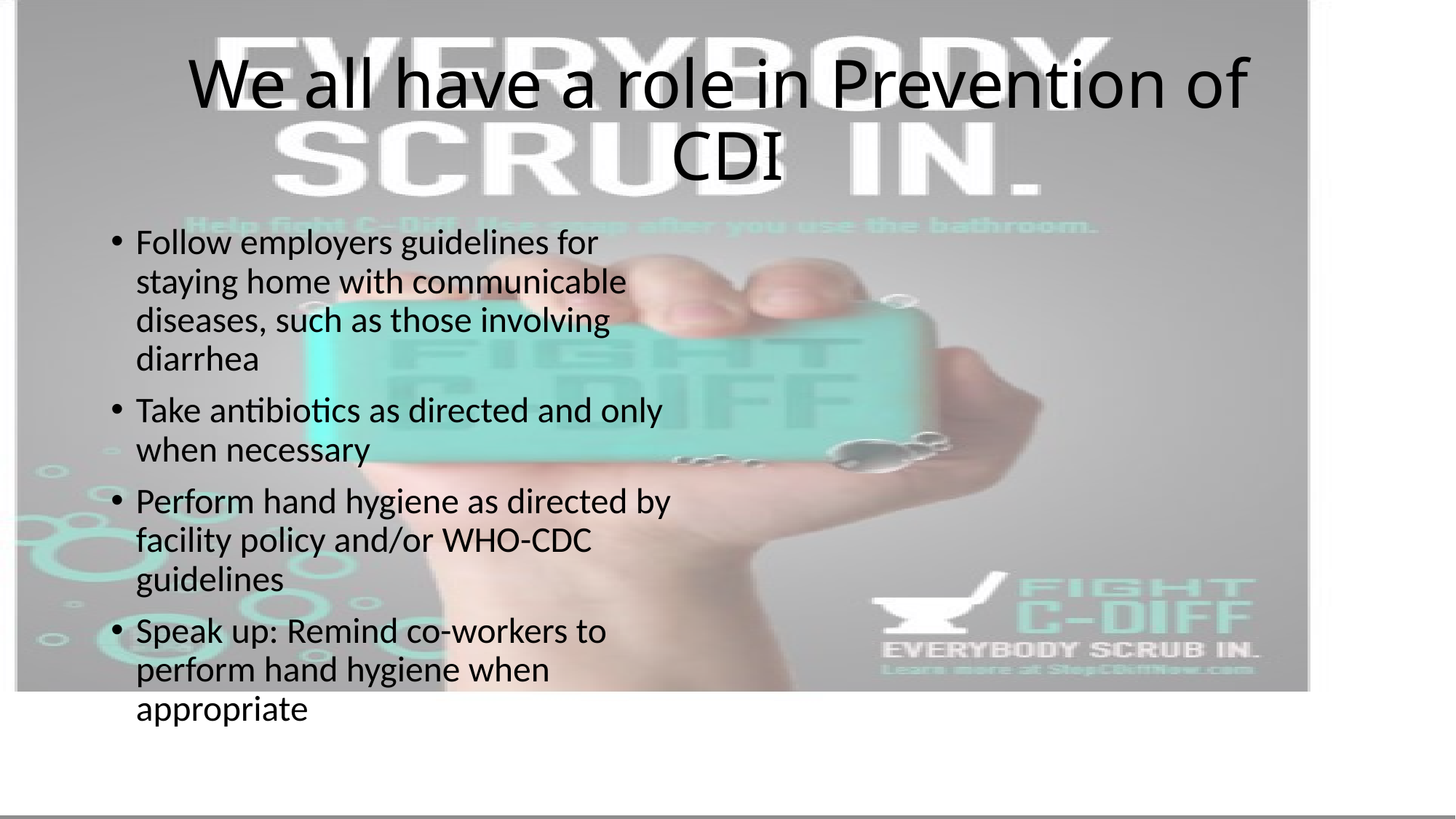

# We all have a role in Prevention of CDI
Follow employers guidelines for staying home with communicable diseases, such as those involving diarrhea
Take antibiotics as directed and only when necessary
Perform hand hygiene as directed by facility policy and/or WHO-CDC guidelines
Speak up: Remind co-workers to perform hand hygiene when appropriate

## Slide 27
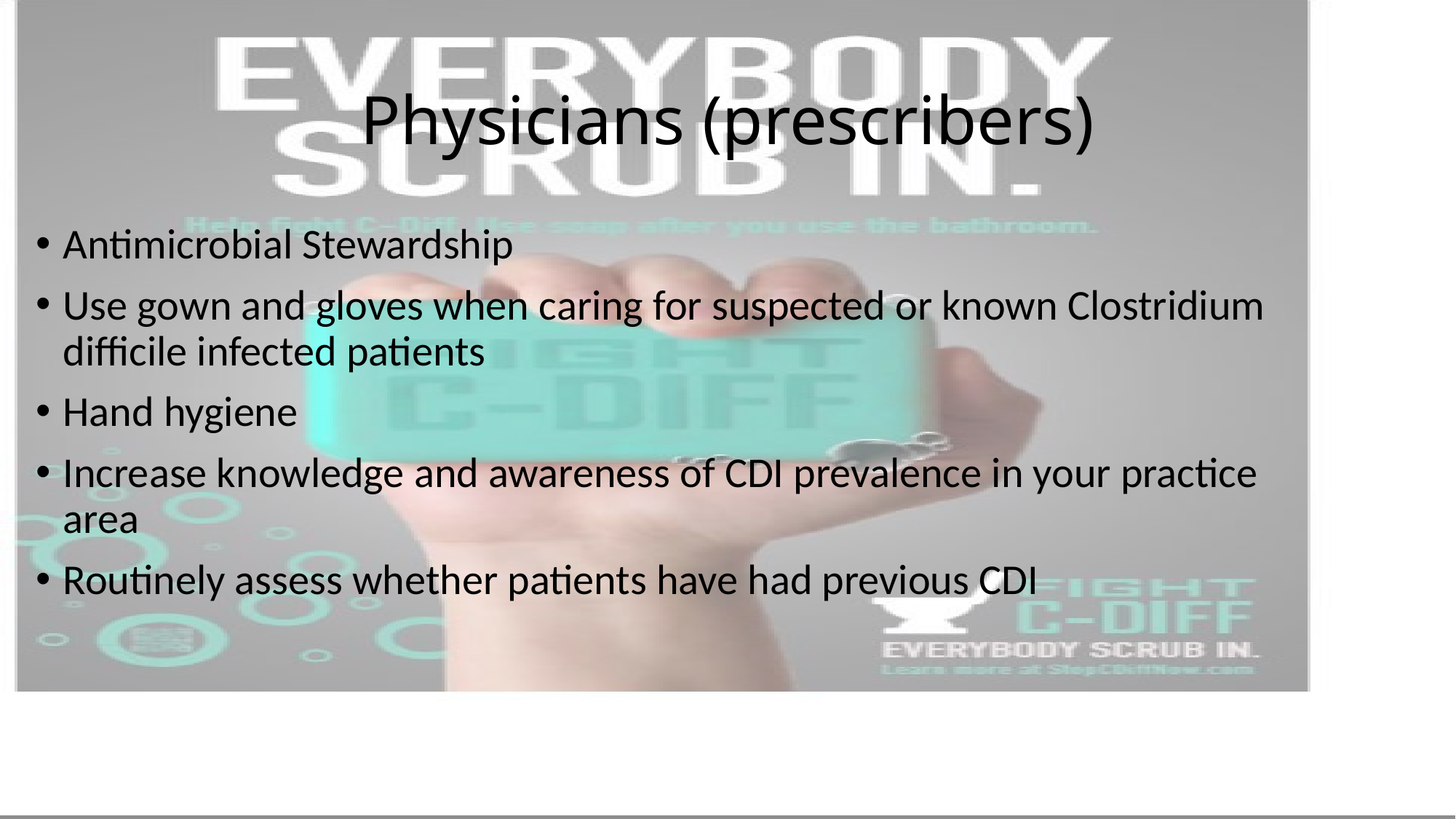

# Physicians (prescribers)
Antimicrobial Stewardship
Use gown and gloves when caring for suspected or known Clostridium difficile infected patients
Hand hygiene
Increase knowledge and awareness of CDI prevalence in your practice area
Routinely assess whether patients have had previous CDI

## Slide 28
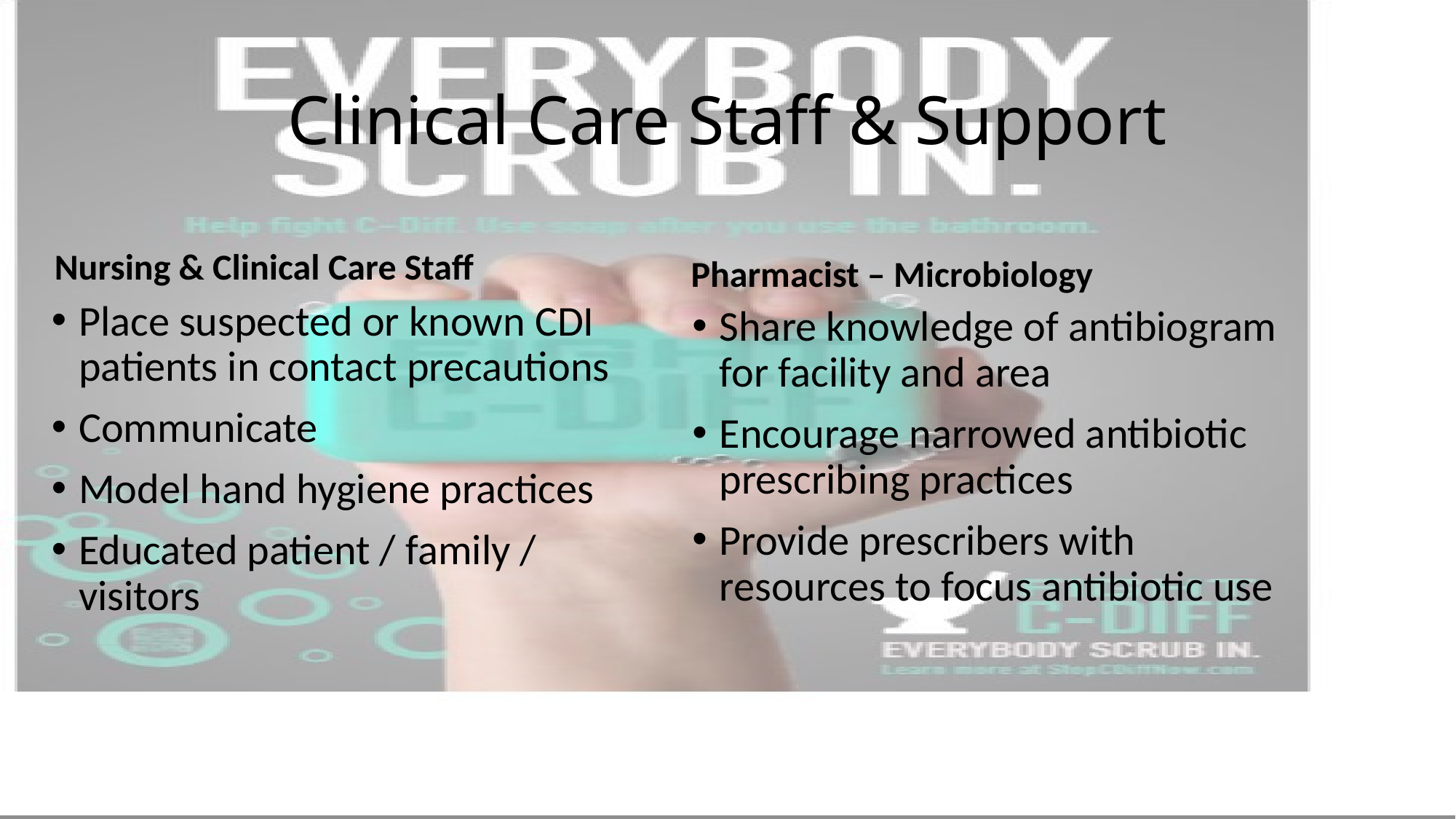

# Clinical Care Staff & Support
Nursing & Clinical Care Staff
Pharmacist – Microbiology
Place suspected or known CDI patients in contact precautions
Communicate
Model hand hygiene practices
Educated patient / family / visitors
Share knowledge of antibiogram for facility and area
Encourage narrowed antibiotic prescribing practices
Provide prescribers with resources to focus antibiotic use

## Slide 29
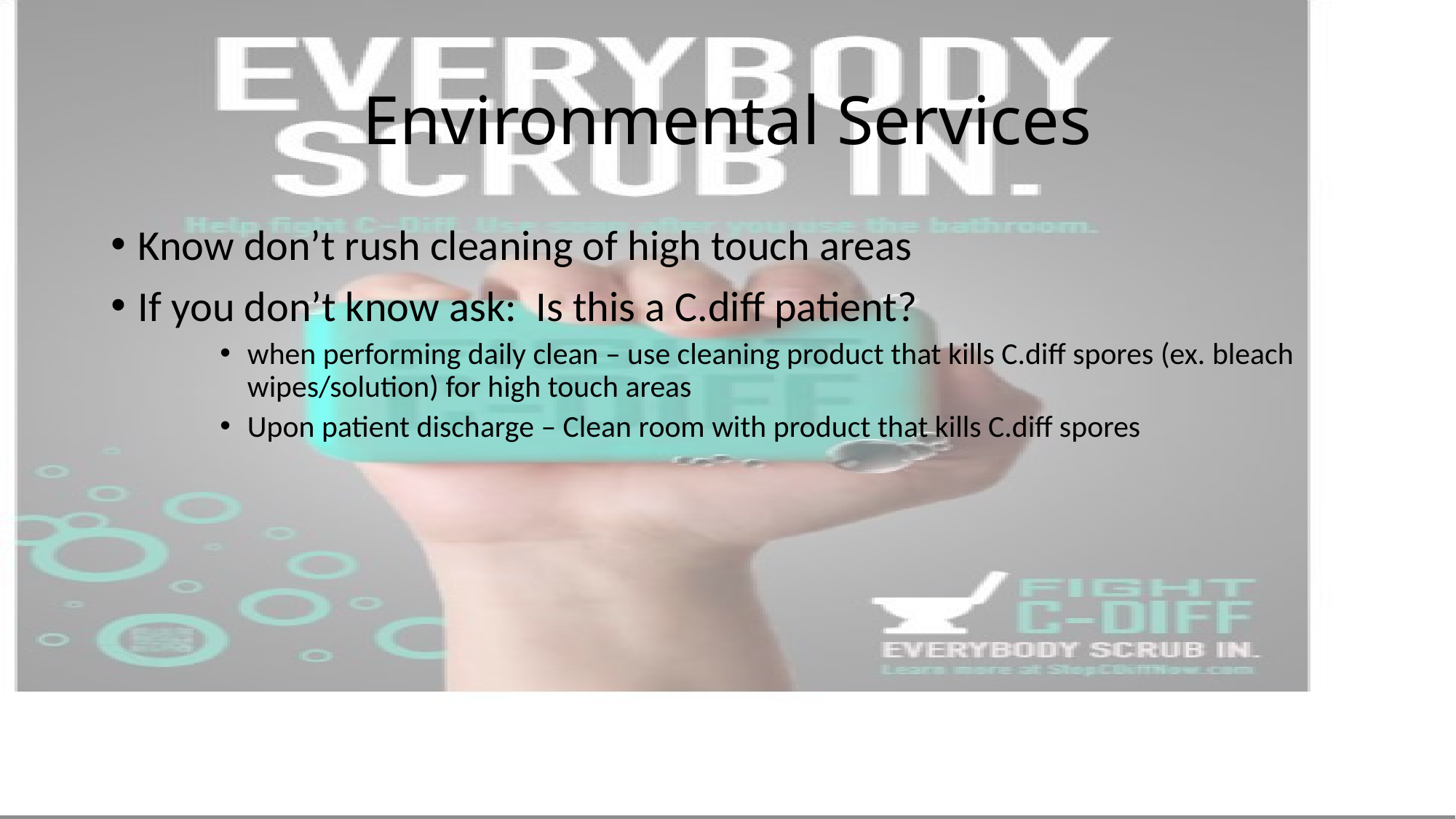

# Environmental Services
Know don’t rush cleaning of high touch areas
If you don’t know ask: Is this a C.diff patient?
when performing daily clean – use cleaning product that kills C.diff spores (ex. bleach wipes/solution) for high touch areas
Upon patient discharge – Clean room with product that kills C.diff spores

## Slide 30
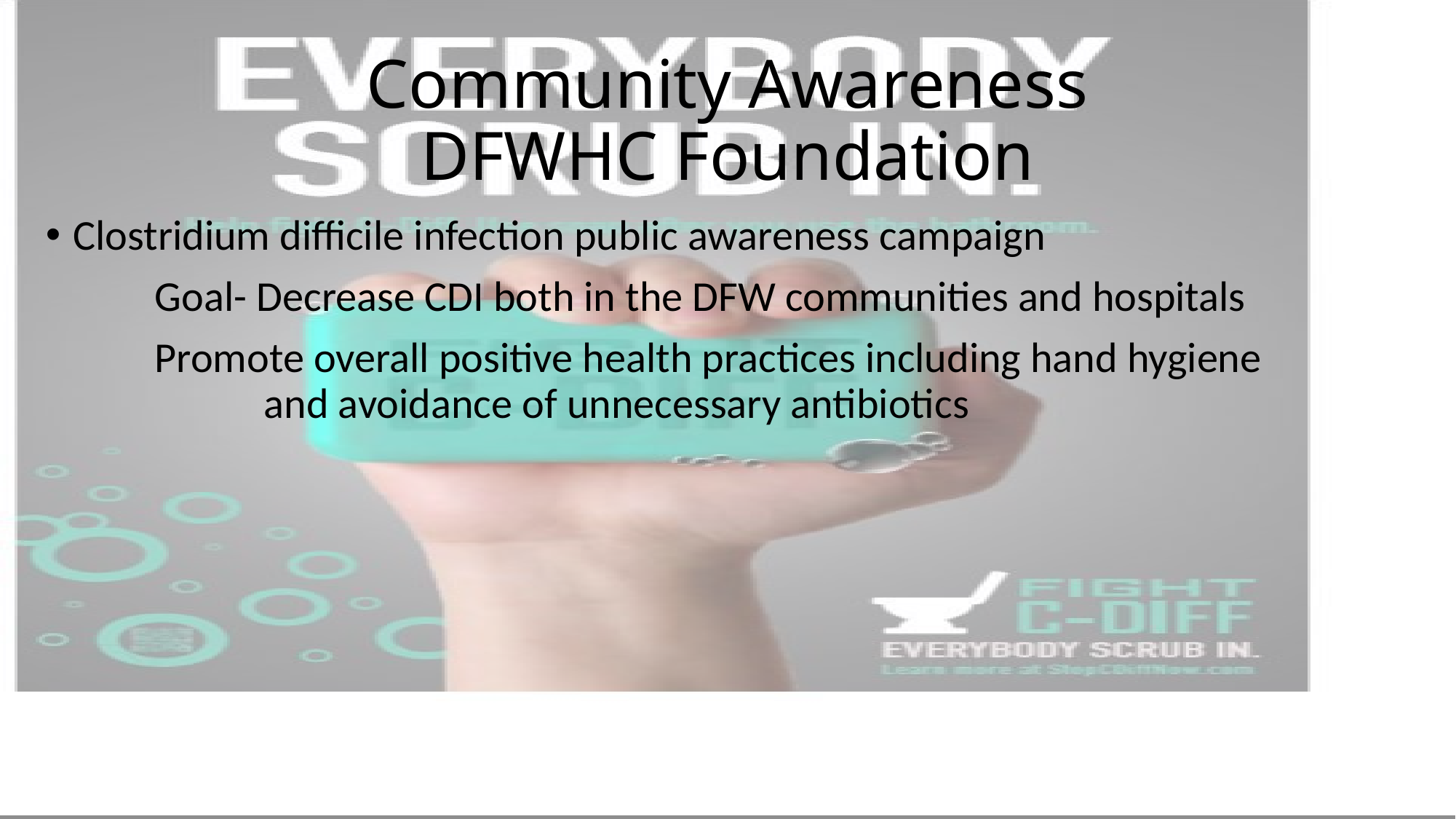

# Community Awareness DFWHC Foundation
Clostridium difficile infection public awareness campaign
	Goal- Decrease CDI both in the DFW communities and hospitals
	Promote overall positive health practices including hand hygiene 		and avoidance of unnecessary antibiotics

## Slide 31
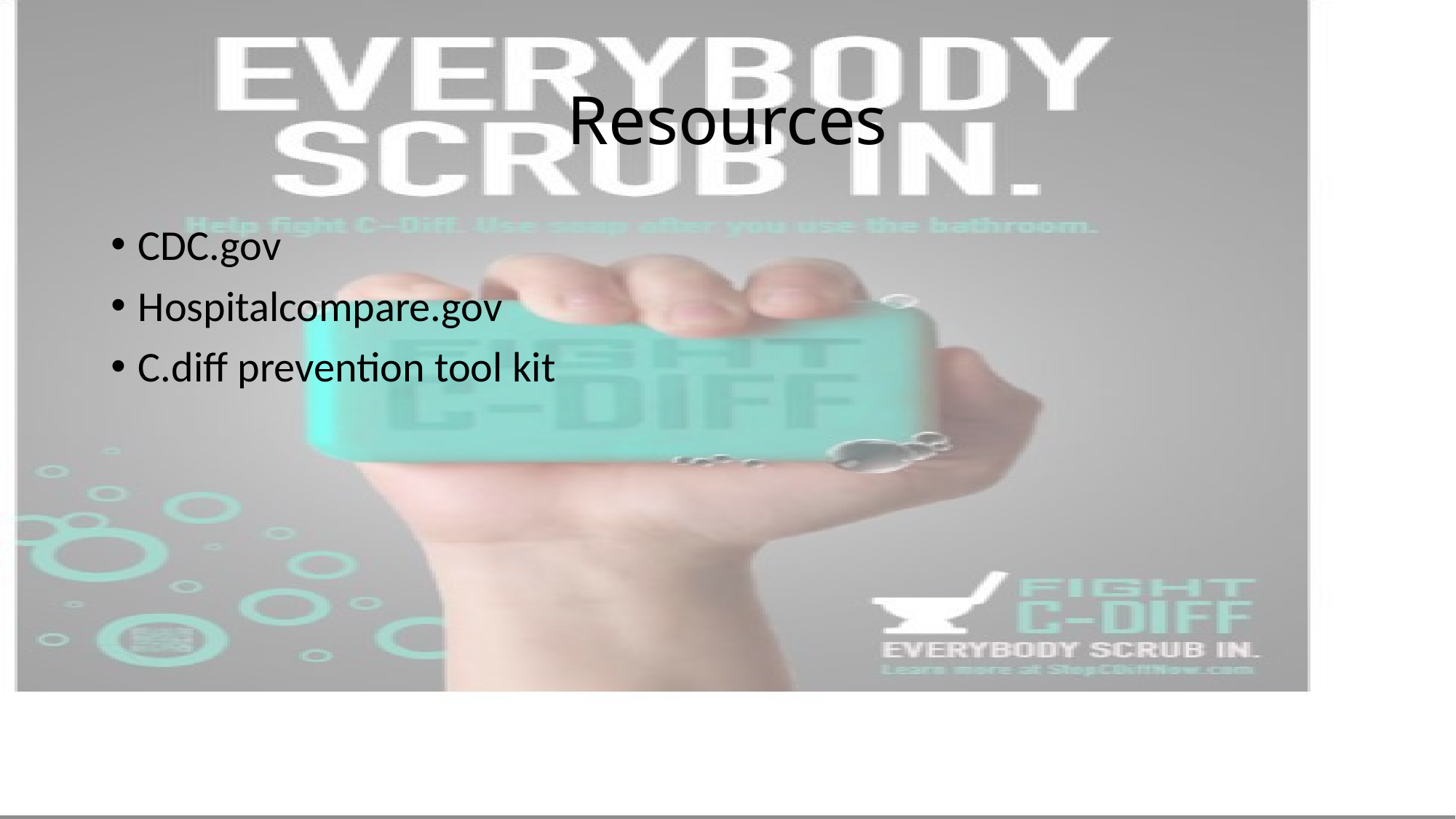

# Resources
CDC.gov
Hospitalcompare.gov
C.diff prevention tool kit

## Slide 32
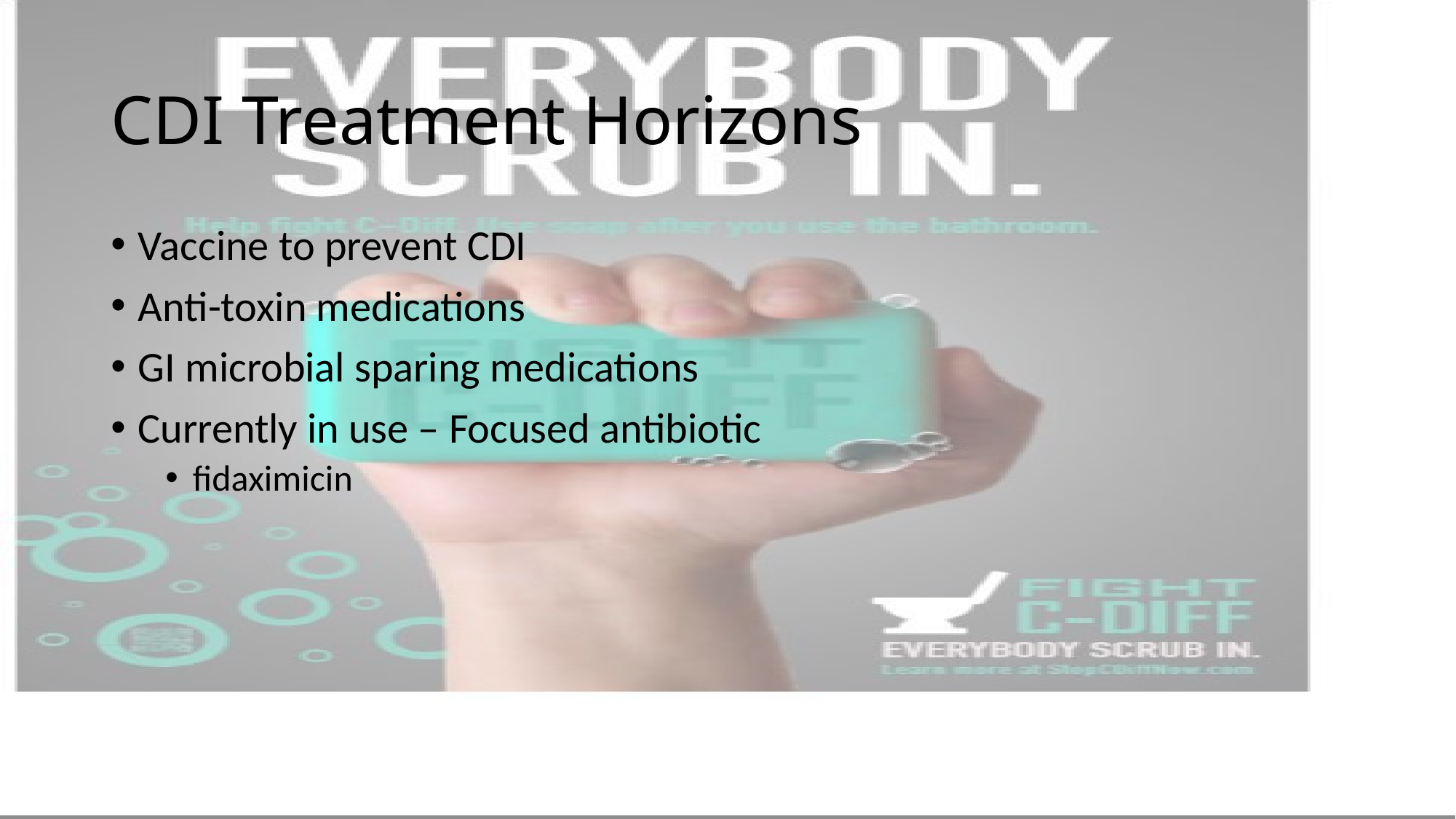

# CDI Treatment Horizons
Vaccine to prevent CDI
Anti-toxin medications
GI microbial sparing medications
Currently in use – Focused antibiotic
fidaximicin

## Slide 33
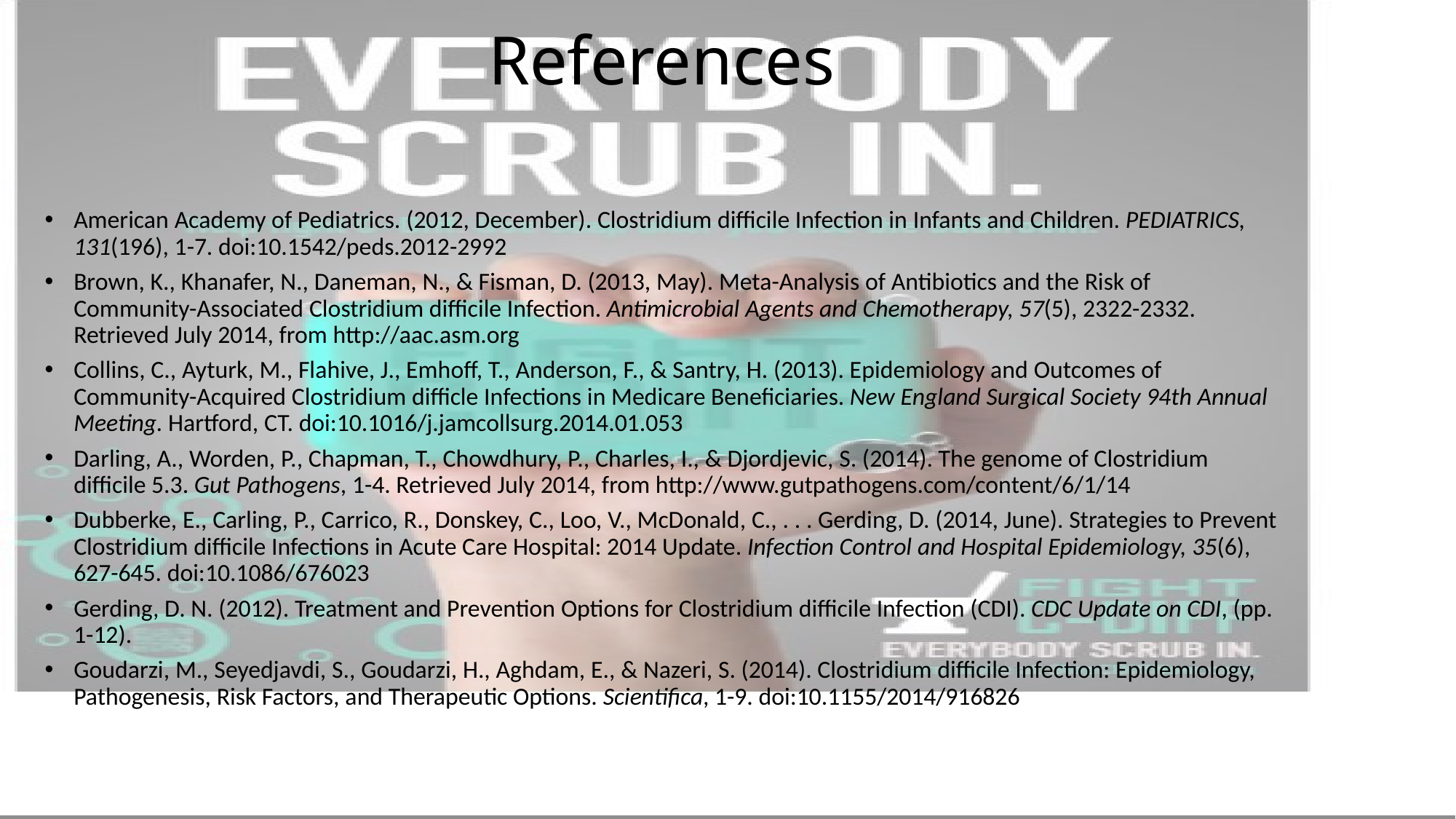

# References
American Academy of Pediatrics. (2012, December). Clostridium difficile Infection in Infants and Children. PEDIATRICS, 131(196), 1-7. doi:10.1542/peds.2012-2992
Brown, K., Khanafer, N., Daneman, N., & Fisman, D. (2013, May). Meta-Analysis of Antibiotics and the Risk of Community-Associated Clostridium difficile Infection. Antimicrobial Agents and Chemotherapy, 57(5), 2322-2332. Retrieved July 2014, from http://aac.asm.org
Collins, C., Ayturk, M., Flahive, J., Emhoff, T., Anderson, F., & Santry, H. (2013). Epidemiology and Outcomes of Community-Acquired Clostridium difficle Infections in Medicare Beneficiaries. New England Surgical Society 94th Annual Meeting. Hartford, CT. doi:10.1016/j.jamcollsurg.2014.01.053
Darling, A., Worden, P., Chapman, T., Chowdhury, P., Charles, I., & Djordjevic, S. (2014). The genome of Clostridium difficile 5.3. Gut Pathogens, 1-4. Retrieved July 2014, from http://www.gutpathogens.com/content/6/1/14
Dubberke, E., Carling, P., Carrico, R., Donskey, C., Loo, V., McDonald, C., . . . Gerding, D. (2014, June). Strategies to Prevent Clostridium difficile Infections in Acute Care Hospital: 2014 Update. Infection Control and Hospital Epidemiology, 35(6), 627-645. doi:10.1086/676023
Gerding, D. N. (2012). Treatment and Prevention Options for Clostridium difficile Infection (CDI). CDC Update on CDI, (pp. 1-12).
Goudarzi, M., Seyedjavdi, S., Goudarzi, H., Aghdam, E., & Nazeri, S. (2014). Clostridium difficile Infection: Epidemiology, Pathogenesis, Risk Factors, and Therapeutic Options. Scientifica, 1-9. doi:10.1155/2014/916826

## Slide 34
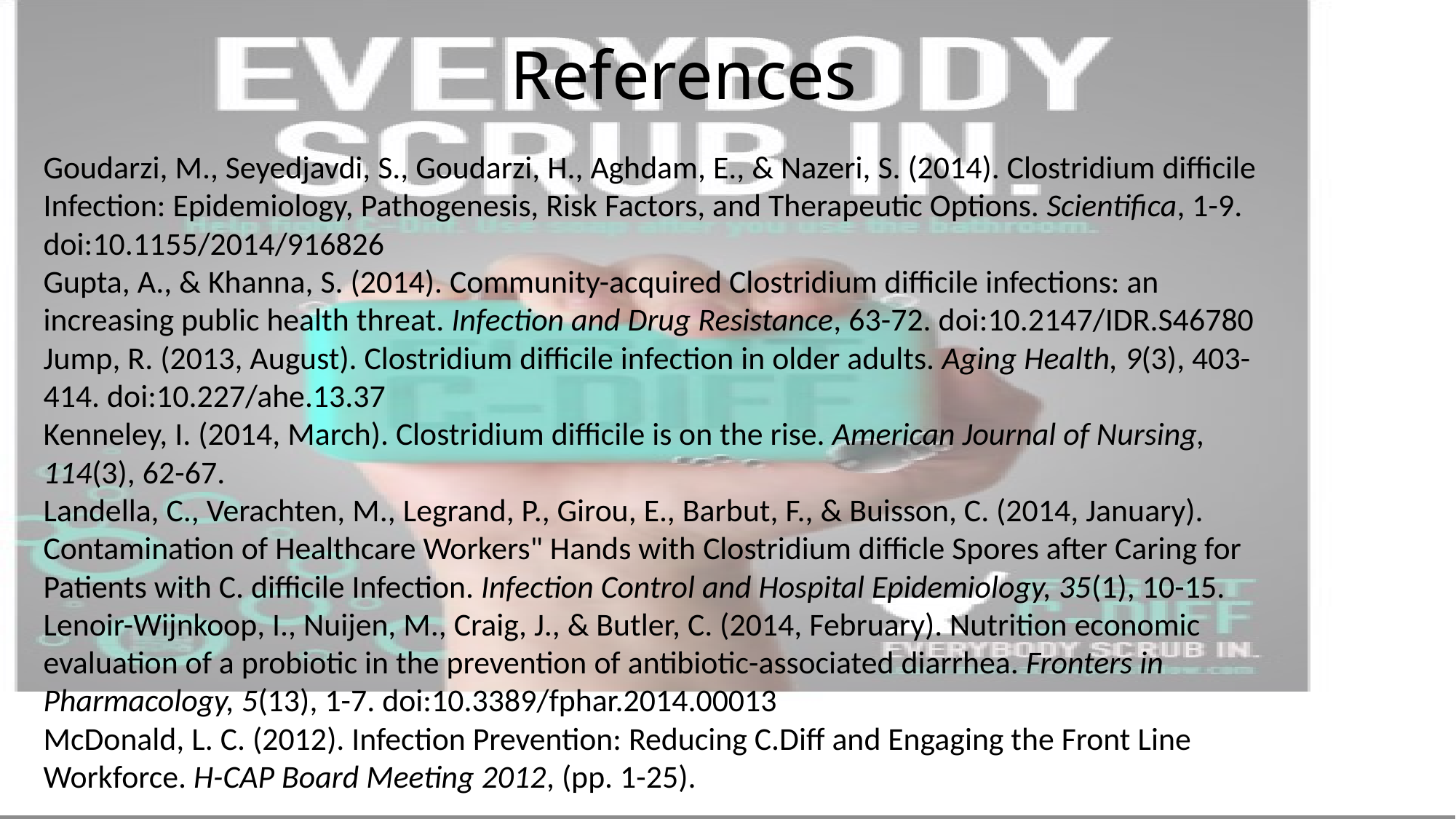

# References
Goudarzi, M., Seyedjavdi, S., Goudarzi, H., Aghdam, E., & Nazeri, S. (2014). Clostridium difficile Infection: Epidemiology, Pathogenesis, Risk Factors, and Therapeutic Options. Scientifica, 1-9. doi:10.1155/2014/916826
Gupta, A., & Khanna, S. (2014). Community-acquired Clostridium difficile infections: an increasing public health threat. Infection and Drug Resistance, 63-72. doi:10.2147/IDR.S46780
Jump, R. (2013, August). Clostridium difficile infection in older adults. Aging Health, 9(3), 403-414. doi:10.227/ahe.13.37
Kenneley, I. (2014, March). Clostridium difficile is on the rise. American Journal of Nursing, 114(3), 62-67.
Landella, C., Verachten, M., Legrand, P., Girou, E., Barbut, F., & Buisson, C. (2014, January). Contamination of Healthcare Workers" Hands with Clostridium difficle Spores after Caring for Patients with C. difficile Infection. Infection Control and Hospital Epidemiology, 35(1), 10-15.
Lenoir-Wijnkoop, I., Nuijen, M., Craig, J., & Butler, C. (2014, February). Nutrition economic evaluation of a probiotic in the prevention of antibiotic-associated diarrhea. Fronters in Pharmacology, 5(13), 1-7. doi:10.3389/fphar.2014.00013
McDonald, L. C. (2012). Infection Prevention: Reducing C.Diff and Engaging the Front Line Workforce. H-CAP Board Meeting 2012, (pp. 1-25).

## Slide 35
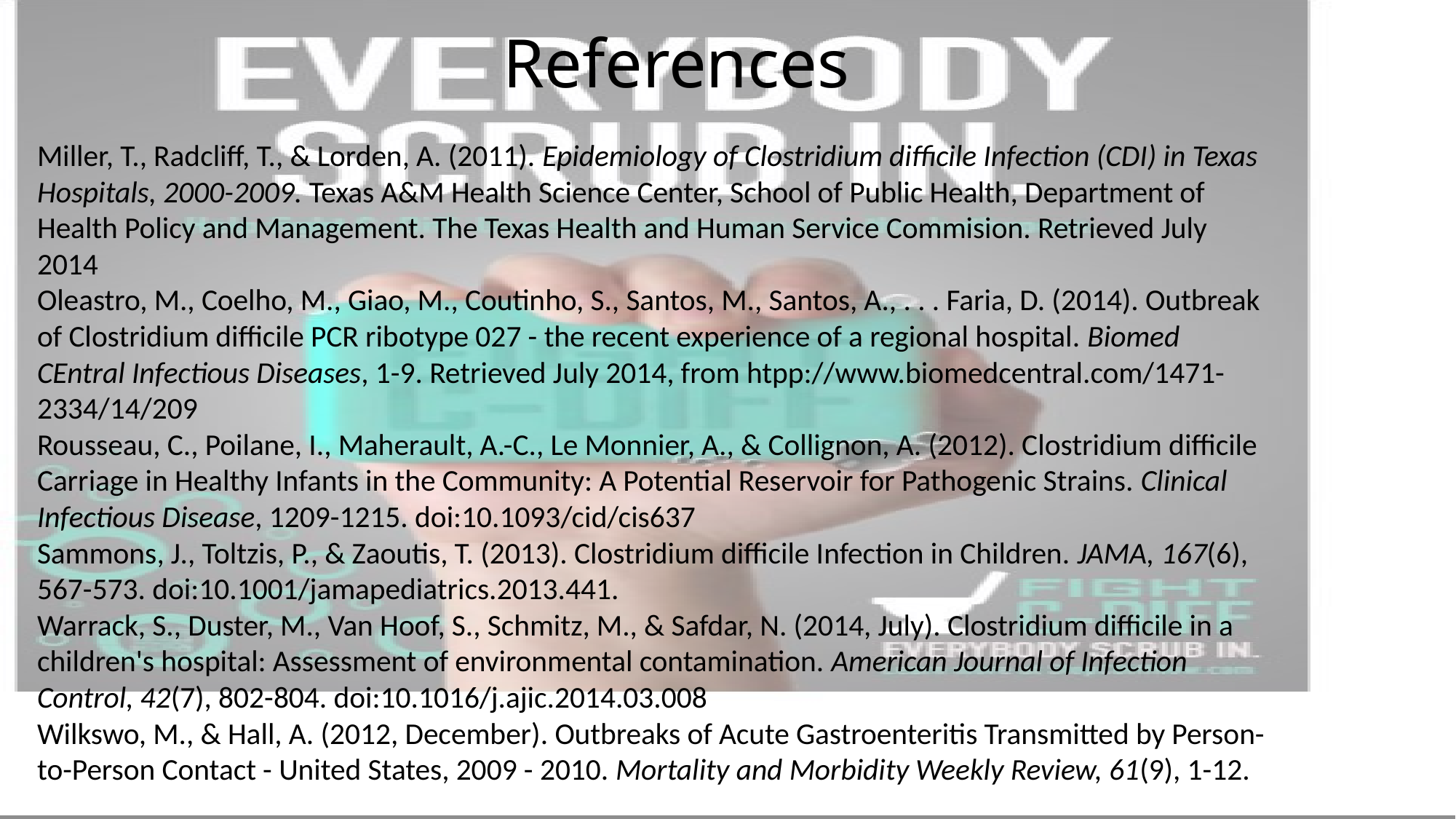

# References
Miller, T., Radcliff, T., & Lorden, A. (2011). Epidemiology of Clostridium difficile Infection (CDI) in Texas Hospitals, 2000-2009. Texas A&M Health Science Center, School of Public Health, Department of Health Policy and Management. The Texas Health and Human Service Commision. Retrieved July 2014
Oleastro, M., Coelho, M., Giao, M., Coutinho, S., Santos, M., Santos, A., . . . Faria, D. (2014). Outbreak of Clostridium difficile PCR ribotype 027 - the recent experience of a regional hospital. Biomed CEntral Infectious Diseases, 1-9. Retrieved July 2014, from htpp://www.biomedcentral.com/1471-2334/14/209
Rousseau, C., Poilane, I., Maherault, A.-C., Le Monnier, A., & Collignon, A. (2012). Clostridium difficile Carriage in Healthy Infants in the Community: A Potential Reservoir for Pathogenic Strains. Clinical Infectious Disease, 1209-1215. doi:10.1093/cid/cis637
Sammons, J., Toltzis, P., & Zaoutis, T. (2013). Clostridium difficile Infection in Children. JAMA, 167(6), 567-573. doi:10.1001/jamapediatrics.2013.441.
Warrack, S., Duster, M., Van Hoof, S., Schmitz, M., & Safdar, N. (2014, July). Clostridium difficile in a children's hospital: Assessment of environmental contamination. American Journal of Infection Control, 42(7), 802-804. doi:10.1016/j.ajic.2014.03.008
Wilkswo, M., & Hall, A. (2012, December). Outbreaks of Acute Gastroenteritis Transmitted by Person-to-Person Contact - United States, 2009 - 2010. Mortality and Morbidity Weekly Review, 61(9), 1-12.

## Slide 36
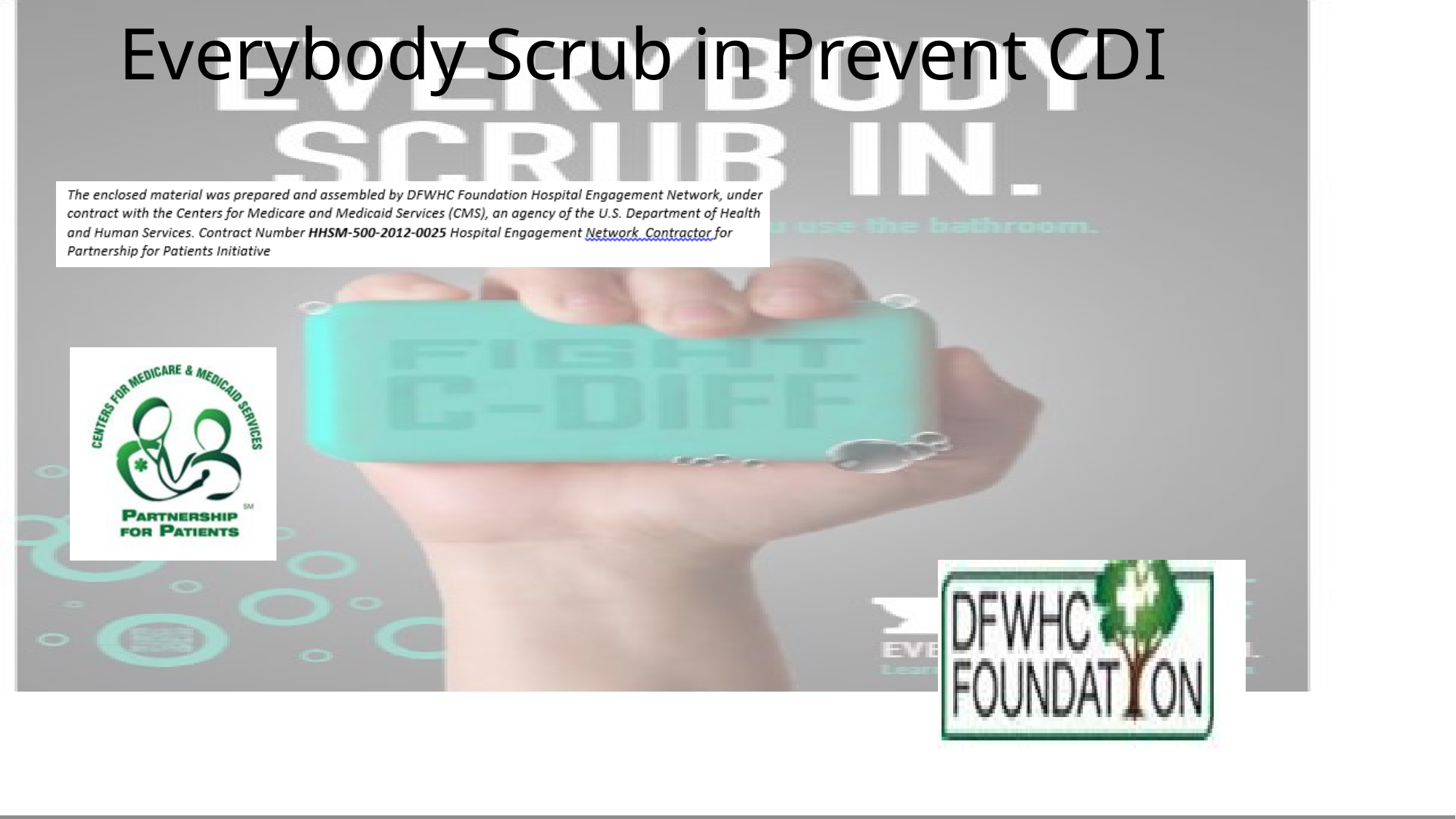

# Everybody Scrub in Prevent CDI
